# The beneficial effects of chick embryo extract preconditioning on hair follicle stem cells: A promising strategy to generate Schwann cells

**DOI:** 10.1111/cpr.13397

**Published:** 2023-01-11

**Authors:** Sareh Pandamooz, Benjamin Jurek, Mehdi Dianatpour, Silke Haerteis, Katharina Limm, Peter J. Oefner, Leila Dargahi, Afshin Borhani‐Haghighi, Jaleel A. Miyan, Mohammad Saied Salehi

**Affiliations:** ^1^ Stem Cells Technology Research Center Shiraz University of Medical Sciences Shiraz Iran; ^2^ Research Group Neurobiology of Stress Resilience Max Planck Institute of Psychiatry Munich Germany; ^3^ Institute of Molecular and Cellular Anatomy University of Regensburg Regensburg Germany; ^4^ Institute of Functional Genomics University of Regensburg Regensburg Germany; ^5^ Neuroscience Research Center Shahid Beheshti University of Medical Sciences Tehran Iran; ^6^ Clinical Neurology Research Center Shiraz University of Medical Sciences Shiraz Iran; ^7^ Faculty of Biology, Medicine & Health, Division of Neuroscience & Experimental Psychology The University of Manchester Manchester UK

## Abstract

The beneficial effects of hair follicle stem cells in different animal models of nervous system conditions have been extensively studied. While chick embryo extract (CEE) has been used as a growth medium supplement for these stem cells, this is the first study to show the effect of CEE on them. The rat hair follicle stem cells were isolated and supplemented with 10% fetal bovine serum plus 10% CEE. The migration rate, proliferative capacity and multipotency were evaluated along with morphometric alteration and differentiation direction. The proteome analysis of CEE content identified effective factors of CEE that probably regulate fate and function of stem cells. The CEE enhances the migration rate of stem cells from explanted bulges as well as their proliferation, likely due to activation of AP‐1 and translationally controlled tumour protein (TCTP) by thioredoxin found in CEE. The increased length of outgrowth may be the result of cyclic AMP response element binding protein (CREB) phosphorylation triggered by active CamKII contained in CEE. Further, CEE supplementation upregulates the expression of vascular endothelial growth factor (VEGF), brain‐derived neurotrophic factor and glial cell line‐derived neurotrophic factor. The elevated expression of target genes and proteins may be due to CREB, AP‐1 and c‐Myc activation in these stem cells. Given the increased transcript levels of neurotrophins, VEGF, and the expression of PDGFR‐α, S100B, MBP and SOX‐10 protein, it is possible that CEE promotes the fate of these stem cells towards Schwann cells.

## Graphical abstract

In this study, chick embryo extract (CEE) was prepared and its proteome content was analysed by tandem mass spectrometry. Treatment of hair follicle stem cells with CEE enhances the migration rate of these stem cells as well as their proliferative capacity likely due to activation of AP‐1 and TCTP by thioredoxin found in CEE. Furthermore, CEE proteome supports these stem cells to acquire neural‐like morphology and increases the expression of proteins that promote their fate towards Schwann cells. Probably, the elevated expression of target genes and proteins is due to activation of CREB, AP‐1 and c‐Myc following CEE preconditioning.

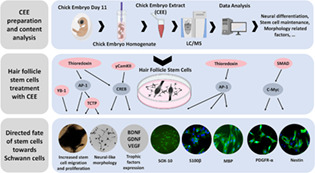

## 1 BACKGROUND

Cell‐based therapy has evolved in several aspects over the last decades and hundreds of clinical trials have been initiated to treat a large panel of pathological indications. Besides the therapeutic effects elicited by the direct presence of stem cells, cell‐derivatives such as extracellular vesicles and other secretome components can also benefit the damaged tissue or organ as critical mediators of trophic factors.^1^ Up until now, various cell sources have been investigated for cell‐based therapies and regenerative medicine.^2^ One of the adult multipotent stem cells that has been considered in the treatment of various neurological conditions is a specific type of hair follicle stem cells called epidermal neural crest stem cells (EPI‐NCSCs). These cells are remnants of the embryonic neural crest,^3^ residing in the bulge of adult hair follicle^4^ that are ontologically related to the central nervous system (CNS) and display a high level of physiological plasticity.^5^, ^6^ These stem cells can differentiate into various cell types, such as neurons^7^, ^8^, ^9^, ^10^, ^11^ and glial cells,^12^, ^13^, ^14^ and express a variety of neurotrophic factors.^15^ Accordingly, they can significantly contribute to the recovery of sensory and motor functions in a mouse and rat model of spinal cord injury.^16^, ^17^, ^18^ In addition, transplantation of hair follicle stem cells supports the repair of peripheral nerve defects^19^, ^20^, ^21^ and has benefits in a rat model of ischemic stroke.^22^, ^23^

Nowadays, several strategies are employed to comply with large‐scale production of stem cells and their derivatives in the most efficient fashion possible to maximize their therapeutic effects. Among introduced approaches, preconditioning has attracted much interest as it increases cell survival rate, differentiation potential, homing and proliferative and secretory capacities of the stem cells.^24^ Preconditioning methods include hypoxia, incubation with trophic factors/cytokines, conditioned medium from functional cells or serum‐free medium, and pretreatment with pharmacological/chemical agents, physical factor assistance and gene manipulation.^25^, ^26^ The pre‐exposure of stem cells to hypoxic conditions triggers various protective signalling pathways, and increases cell survival and cytokine secretion that can greatly improve the benefit of in vivo stem cells therapy.^27^ Using pharmacological/chemical agents, such as various off‐label drugs, is another promising approach that can optimize the restorative potential of transplanted stem cells.^28^ The modulation of the biochemical and biophysical microenvironment, mediated by either naturally derived or synthetic biomaterials, is another way to influence stem cell fate and to enhance their therapeutic potential.^29^ An additional strategy to achieve successful therapeutic outcomes is the genetic modification that induces overexpression or knock out/down of a certain gene to improve the stem cells' inherent restorative properties.^30^ Although genetically engineered stem cells are widely used in pre‐clinical investigations, there are lots of limitations and unsolved issues hampering their clinical application.^31^ In addition, priming of stem cells with different cytokines and growth factors is commonly used to stimulate the secretion of anti‐inflammatory and immunomodulatory factors and to improve the immunosuppressive functions of stem cells to withstand the harsh microenvironment of target tissues. However, a major challenge of using this preconditioning tool is the high costs of priming with recombinant cytokines and trophic factors.^32^

Thus far, several preconditioning strategies including pharmacological/chemical agents^13^, ^15^ as well as substrate stiffness priming^33^ have optimized the functionality of EPI‐NCSCs in vitro. This study was designed to assess the chick embryo extract (CEE) preconditioning effect on the fate and function of EPI‐NCSCs in culture. CEE is a complex cocktail of growth factors that has been widely used to supplement the growth media of various cell types, such as neural crest stem cells (NCSCs).^34^, ^35^, ^36^ In the present study, CEE was collected and used to determine whether its presence can stimulate migration of stem cells from the hair bulge, proliferation rate of migrated stem cells, and alter their morphology. Also, the influence of CEE treatment on expression of key cell surface markers, differentiation into osteoblast and adipocyte as well as colony‐forming efficiency were assessed. The potential of stem cells to form the three‐dimensional (3D) structure of spheroids was another subject that was evaluated following CEE preconditioning. In addition, analysis of gene expression involved in the fate and secretion of trophic factors was performed using reverse transcription (RT)‐polymerase chain reaction (PCR). Following gene expression analysis, immunostaining against specific lineage markers was carried out to define stem cells commitment. To identify CEE relevant proteins that possibly affect migration, proliferation, gene and protein expression, and morphological alteration of hair follicle stem cells, the proteome content of CEE was analysed by tandem mass spectrometry.

Indeed, the beneficial role of hair follicle stem cells to reduce pathological indications and improving tissue repair has been proven in animal models of various neurological conditions. We, therefore, sought to investigate if preconditioning of these stem cells with CEE could help to acquire some desirable traits that can enhance their therapeutic benefit in preclinical studies of central and peripheral nervous system damage. We, thus provide first evidence that CEE preconditioning can promote lineage commitment of hair follicle stem cells and owing to specific proteome content might improve transplantation success.

## 2 METHODS

### 2.1 Preparation of CEE

To prepare CEE, 11‐day‐old fertilized chick eggs were disinfected using 70% ethanol. The embryos were removed from the shell and washed with ice cold Hanks balanced salt solution, their heads were cut off and the rest of the embryo was chopped and centrifuged as previously described.^35^, ^37^ Finally, the extract was filtered through 0.45 and 0.22‐μm filters and samples were stored at −80°C until use. The sterility test for bacterial, fungi and yeast contamination revealed the samples are free from contamination.

### 2.2 Isolation, in vitro expansion and treatment of hair follicle stem cells

Hair follicle stem cells were obtained from the bulge of rat whisker pads hair follicles as previously described.^38^ Briefly, the hair follicles were mechanically dissected from rat whisker pads and the bulge region was excised and explanted onto collagen (1 mg/mL, Roche; #11179179001) coated 4‐well plates. The explanted bulges were fed with alpha‐modified minimum essential medium (α‐MEM, Bio Idea, # BI‐1010‐05) that was supplemented with 1% penicillin/streptomycin (Bio Idea, # BI‐1203), 1% L‐Glu (Bio Idea, # BI‐1202) and either 10% fetal bovine serum (FBS, Bio Idea, # BI‐1201) (experimental group 1, i), 20% FBS (experimental group 2, ii) or 10% FBS plus 10% CEE (experimental group 3, iii). The bulges were incubated in a cell culture incubator at 37°C, 5% CO_2_. Following stem cell migration, cells were subcultured at nearly 80% confluency, using 0.25% Trypsin/EDTA (Bio‐Idea, # BI‐1602). In this study, the images of explanted bulges of different experimental groups at different days of in vitro culture (DIV: 3, 5, 7 and 11) were captured with a ZOE Fluorescent Cell Imager (Bio‐Rad). To compare the number of migrated stem cells between groups, cells were counted following first subculture, using trypan blue staining.

### 2.3 Cell viability assay

To assess cellular viability, the 3‐(4,5‐Dimethylthiazol‐2‐yl)‐2,5‐Diphenyltetrazolium Bromide (MTT) assay was performed after third subculture. Cells from the different experimental groups were seeded in a 96‐well plate at an equal density of 10^4^ cells per well and cultivated in their respective complete growth medium at 37°C in the CO_2_ incubator. On the third day, the medium was discarded and 0.5 mg/mL MTT (Sigma‐Aldrich, Cat No: M5655) prepared in α‐MEM was added to each well. After 3 h incubation, the MTT solution was gently aspirated and acidic isopropanol (0.01 N HCl in absolute isopropanol, 100 μl/well) added to dissolve the blue formazan crystals. The developed colour was measured at 570 nm using a microplate reader (BioTek).

### 2.4 Colony‐forming assay

To compare colony‐forming efficiency between experimental groups, an in vitro colony formation assay was performed. First, 300 cells were seeded into 6‐cm plates and cultured with their corresponding growth medium for 10 days. The colonies were fixed with 4% paraformaldehyde (PFA) and stained with crystal violet. The number of colonies and their sizes were defined for each individual plate and analysed with Prism software. This experiment was performed in triplicate and repeated trice.

### 2.5 Flow cytometry

The immunophenotype of cell surface markers in each experimental group was assessed at P3 with fluorochrome‐conjugated antibodies to CD44 (CD44‐FITC, BioLegend, # 203906), CD90 (CD90‐PerCPCY5.5, BioLegend, # 202515), CD34 (CD34‐PE, BDBioscience, #553142) and CD45 (CD45‐FITC, BDBioscience, #550616). The assay was performed using a BD FACSCalibur (BD, Biosciences) and final histogram was generated with FlowJo v10 software (FlowJo LLC).

### 2.6 Differentiation assay

Osteogenic differentiation of cells in different experimental groups was induced by culturing of the cells in OsteoPlus differentiation medium (Bioidea, #BI‐1102) for 21 days. Also, lipogenic differentiation was conducted in AdipoPlus medium (Bioidea, #BI‐1101) for 21 days, as well. To confirm osteogenic and adipogenic differentiation, cells were fixed for 20 min in 4% PFA and stained with Alizarin Red (Bioidea, #BI‐1801) to detect calcified extracellular matrix deposits or Oil Red O (Bioidea, #BI‐1802) to stain neutral lipids. After washing, images were collected on an Olympus IX71 inverted microscope (Olympus) coupled with an Olympus DP25 camera. In parallel, the transcript level of genes related to osteogenic and adipogenic differentiation was evaluated following 21 days of induction.

### 2.7 Alkaline phosphatase activity assay

To measure alkaline phosphatase (ALP) activity, as a reliable marker for osteogenic differentiation, cells from different experimental groups were seeded in 6‐well plate and incubated with osteogenic medium. After 21 days of induction, cells were lysed with RIPA buffer (Kiazist, #KRIP100) containing protease inhibitor cocktail (KPICM), followed by centrifugation. The ALP activity was defined with the ALP assay kit (biorexfars, #BXC0187) according to the manufactures' instruction, and normalized to total protein concentration of each sample measured with BCA assay kit (Kiazist, # KBCA96).

### 2.8 Immunofluorescent staining

To verify the identity of migrated stem cells and their fate following CEE treatment, immunofluorescent staining was performed. To do so, cells of each experimental group were seeded in 4‐chamber glass slides at a density of 7.5 × 10^4^ cells per chamber. The following day, 4% PFA was added to the cell culture medium (1:1) for 2 min, the medium was aspirated and 1 ml of 4% PFA was added to each well for another 10 min. Following three washes with PBS‐T, cells were blocked with blocking solution (0.1% Triton X‐100, 1% FBS, 10% normal goat serum prepared in PBS) for 30 min and incubated in diluted primary antibody solution (0.5% Triton X‐100, 3% FBS prepared in PBS) overnight at 4°C. Primary antibodies were used as follows: mouse anti‐nestin monoclonal antibody (1:50; Abcam, #ab6142), rabbit anti‐β‐tubulin polyclonal antibody (1:50, Cell Signalling, #2146s), rabbit anti‐SOX‐10 polyclonal antibody (1:100, Proteintech, #10422‐1‐AP), rabbit anti‐βII‐tubulin polyclonal antibody (1:200, Abcam, #ab18207), AlexaFluor488 Phalloidin (1:200, Cell Signalling, #cs8878), chicken anti‐MAP2 polyclonal antibody (1:500, Synaptic Systems, #188006), rabbit anti‐GFAP monoclonal antibody (1:500, Cell Signalling, #12389s), rabbit anti‐PDGFRα (1:200, Abcam, #ab61219), mouse anti‐S100 B (β‐subunit) monoclonal antibody (1:500, Sigma‐Aldrich, #S2532), rabbit anti‐Myelin Basic polyclonal antibody (1:200, Abcam, #ab40390), rabbit anti‐ SOX10 polyclonal antibody (1:100, Proteintech, #10422‐1‐AP), and rabbit anti‐SOX10 monoclonal antibody (1:250, Abcam, #ab155279). The next day, cells were washed thrice with PBS‐T, re‐blocked with 3% BSA for 10 min, and incubated with corresponding secondary antibodies: goat anti‐mouse IgG AlexaFluor488 (1:1000, ThermoFisher, #A‐11001), goat anti‐rabbit IgG AlexaFluor488 (1:1000, ThermoFisher, #A‐11008), and goat anti‐chicken IgG AlexaFluor 488 (1:1000, Invitrogen, #A11039), for 2 h at room temperature. Finally, each chamber was covered with one drop of ProLong™ Glass Antifade Mountant with NucBlue™ Stain (Invitrogen, # P36985) to counterstain the nuclei and a coverslip was used to avoid sample drying. Images were captured with a Leica DM5000B epifluorescence microscope.

### 2.9 Morphological assessment

To determine the number of cells with neurites as well as the number of neurites per cell and their length, cells were seeded on a 12‐well plate at a density of 1.5 × 10^5^ cells/well and incubated overnight. Then, cells were gently fixed by adding 1 ml 4% PFA to each well. After 2 min, this solution was discarded and replaced with 4% PFA for another 10 min. The cells were washed three times with PBS and stained with crystal violet solution and images were captured using ZOE fluorescent cell imager (Bio‐Rad). Here, counting of cells with neurites and number of neurites per cell was performed manually. To this aim, the number of cells with neurites was counted in 33 randomly selected captured images of each experimental group (total 70 captured images). Then the percentage of cells with 1 to maximum 6 neurites per cell was counted. Neurite length was also measured manually by tracing the length of the longest neurite per cell from the edge of the nucleus to the tip of the projection (using the measuring tool in ImageJ Fiji version 1,52r software, NIH, USA).

### 2.10 Spheroid formation assay

To assess the ability of these stem cells to form spheres in different experimental groups, following second subculture, the trypsinized stem cells were seeded on 1% agarose coated 96‐well plates at a density of 5 × 10^3^ cells in a volume of 200 μl per well, as previously described.^39^

### 2.11 RNA isolation and quantitative RT‐PCR

Total RNA was extracted from cells grown in 6‐well plates at ~90% confluency, using YTzol (Cat. No: YT9063; Yekta Tajhiz Azma, Iran) reagent by chloroform extraction and ethanol precipitation, according to the manufacturer's instructions, followed by measurement of total RNA concentration and purity using NanoDrop spectrophotometer (Thermo Scientific). cDNA was synthesized from 1 μg total RNA using a reverse transcriptase kit (Cat No: YT4500; Yekta Tajhiz Azma). Then, real time‐PCR was performed in triplicate using RealQ Plus 2x Master Mix Green (Cat. No: A325402; Ampliqon, Denmark) and primers (listed in Table 1) on a ABI StepOne Real‐Time PCR system (Applied Biosystems). The reaction cycle consisted of 95°C for 15 min, followed by 40 cycles of 95°C for 15 s, and 60°C for 1 min. The reference gene HPRT was employed as internal reference control. The 2^−ΔΔCt^ method was used to calculate the relative changes in expression of the target genes.

**TABLE 1:** List of primers and their respective amplicon length in this experiment

Gene	Forward primer (5′‐3′)	Reverse primer (5′‐3′)	Amplicon length (bp)
ALP	CCTGACTGACCCTTCCCTCTC	CAATCCTGCCTCCTTCCACTA	101
Runx2	CGCACGACAACCGCACCAT	CTCTCCGAGGGCTACAACCT	155
BMP2	GAGTTTGAGTTGAGGCTGCT	GATGGCTTCTTCGTGATGGA	201
PPAR‐γ	AGTGGGAATTAAGGCAA	CACCATGCTCTGGGTCAA	89
Nestin	CAAGGTCTGGTCTGGTGTATGC	GCTTTATTCAGGGAGGAAGAGAGG	106
DCX	CGCCGCAGCAAGTCTCCAG	TCGCCAAGTGAATCAGAGTCATCC	185
SOX‐10	ACGCAGAAAGTTAGCCGACCAG	CACTCTCGTTCAGCAACCTCCAG	92
β‐III tubulin	GCTGGAACGCATCAGTGTCTAC	GCACCACTCTGACCGAAGATAAAG	162
GFAP	GGGACAATCTCACACAGGACCTC	CCTCCAGCGACTCAACCTTCC	162
PDGFR‐α	AACCGAGGAGAACAACAGTAGCC	AAGAATCCGTCATGCCGAGAGG	194
MAP2	AAGCGGAAAACCACAGCAACAAG	TTCTCCTCCCTGTCTCCTGATACG	176
BDNF	CGATTAGGTGGCTTCATAGGAGAC	CAGAACAGAACAGAACAGAACAGG	182
GDNF	GCTGACCAGTGACTCCAATATGC	CCTCTGCGACCTTTCCCTCTG	192
VEGF	ACTTGAGTTGGGAGGAGGATGTC	GGATGGGTTTGTCGTGTTTCTGG	183
HPRT	CCAGCGTCGTGATTAGTGATGATG	GAGCAAGTCTTTCAGTCCTGTCC	135

### 2.12 Proteome analysis of CEE content

Freshly prepared CEE was flash‐frozen and subjected to proteome analysis as previously described.^40^ Briefly, proteins were extracted and trypsinated using gel‐assisted sample preparation. Resulting peptides were separated by liquid chromatography prior to peptide sequence analysis by tandem mass spectrometry in data‐dependent acquisition mode on an AB Sciex TripleTOF 5600+. For protein identification, sequence data were blasted against both, the non‐curated UniProtKB/TrEMBLE and the manually annotated and non‐redundant UniProtKB/Swiss‐Prot database.

### 2.13 Statistical analysis

Statistical analysis was performed using GraphPad Prism (Version 7.03, GraphPad Software Inc.). One‐ and two‐way analysis of variance with Tukey post hoc correction were performed, and, where appropriate, *t*‐test to detect statistical differences among the experimental groups. *p* < 0.05 was considered to be statistically significant. The data are presented as means ± SEM.

## 3 RESULTS

### 3.1 Characterization of EPI‐NCSCs

**FIGURE 1:**
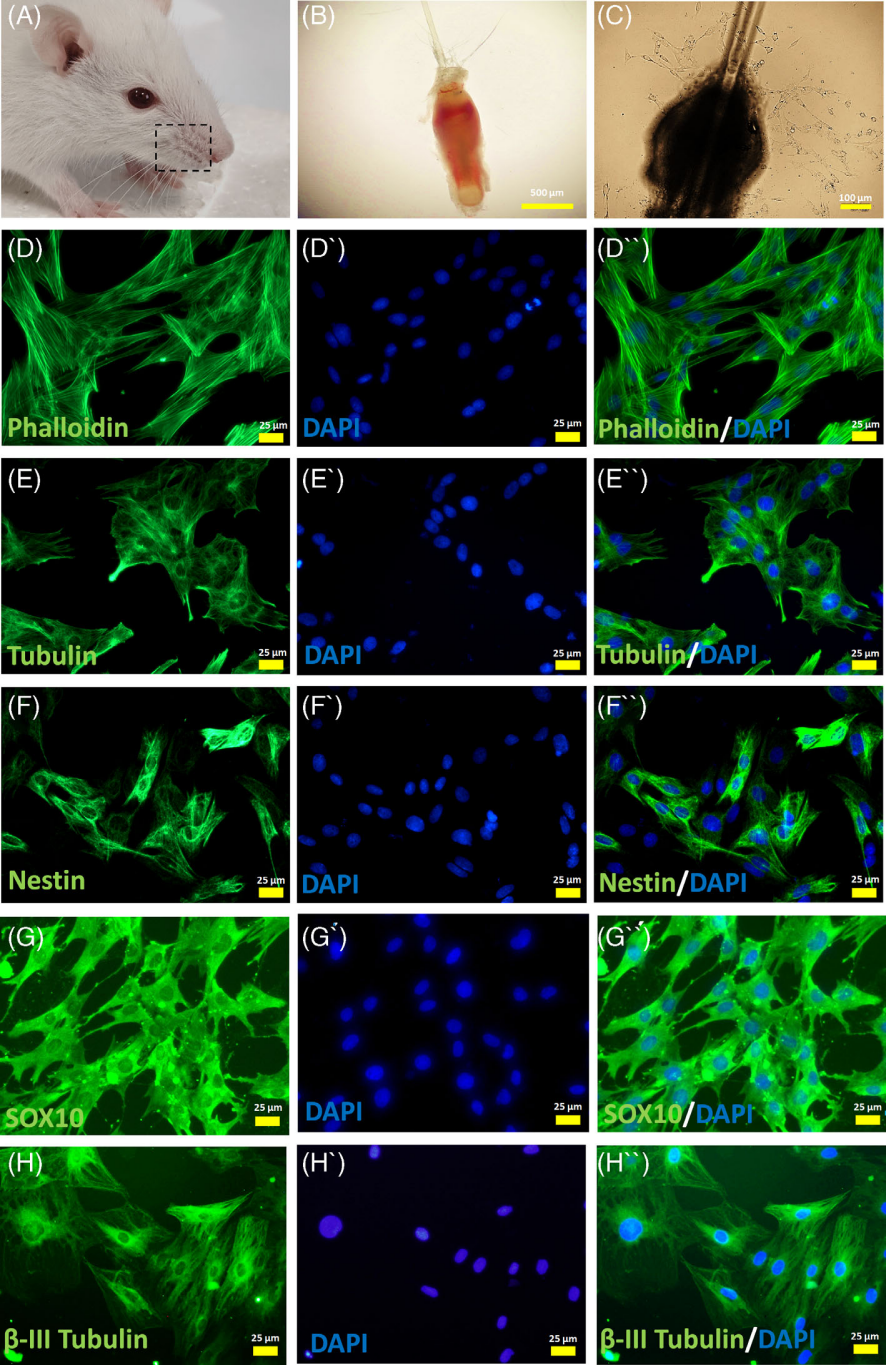
Isolation and characterization of rat hair follicle stem cells. (A, B) In the currrent study, the whisker pads of rat was dissected to isolate large hair follicles. Then, the bulge region of the hair follicle was carefully microdissected and explanted onto collagen coated plates. (C) Few days later, migrated stem cells were detected around the explanted bulge. (D,E) The immunostaining against phalloidin and β‐Tubulin revealed the general morphology of stem cells. (F‐H) Nestin, SOX‐10 and β‐III Tubulin expression confirmed the identity of migrated cells as hair follicle stem cells. Cell nuclei were counterstained with DAPI. Images are examples of three different assessments for each immunostaining (*n* = 3)

A few days after bulge isolation from whisker pads (Figure 1A,B) and explantation on collagen coated wells, migrating stem cells with stellate morphology were observed around the bulges in all primary experimental groups (i, ii, iii) (Figure 1C). Phalloidin conjugated to Alexa Fluor 488 was used for staining filamentous actin (F‐actin) (Figure 1D), while immunostaining of β‐tubulin revealed the general morphology of migrating stem cells (Figure 1E). Also, indirect immunofluorescent staining against Nestin (marker of NCSCs) (Figure 1F) and other NCSC markers, such as SOX‐10 (Figure 1G), β‐III Tubulin (Figure 1H) and GFAP (data not shown) verified the identity of migrating cells as EPI‐NCSCs.^14^, ^33^ It is worth noting, that representative immunostaining micrographs were provided from CEE supplemented experimental group after the first subculture.

### 3.2 EPI‐NCSC migration and expansion enhanced in presence of CEE

**FIGURE 2:**
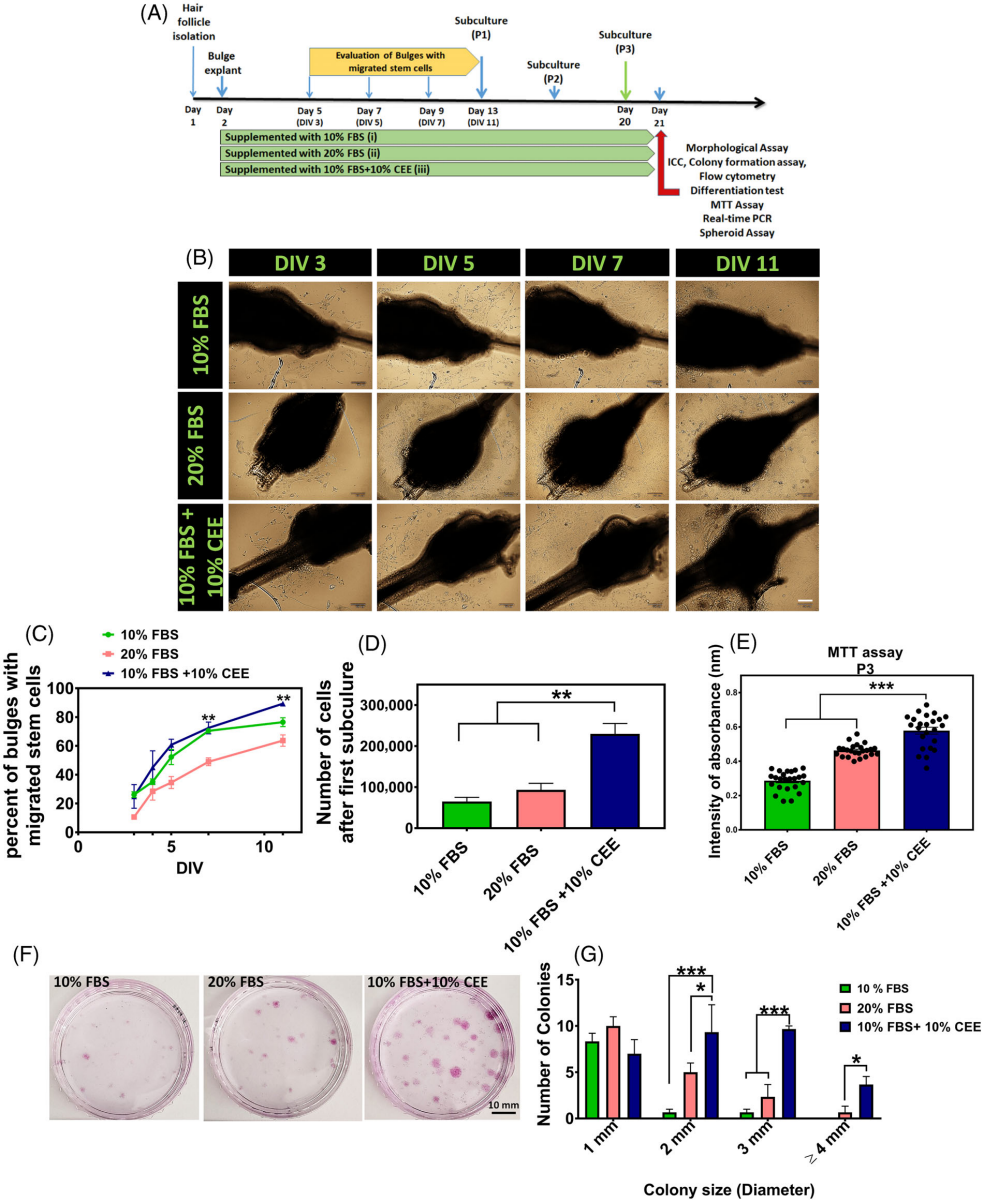
Epidermal neural crest stem cells in vitro migration and expansion. (A) Schematic time‐line protocol of the study. (B) In this study, the explanted bulges were supplemented with different concentrations of fetal bovine serum (FBS) and chick embryo extract (CEE). Evaluation of the bulges at DIV: 3, 5, 7 and 11 revealed increasing rates of migrating stem cells around the bulges in all experimental groups, Scale bar: 100 μm. (C) Although the percent of bulges with migrated stem cells increased over the culture time course in all three groups, it was significantly higher in experimental group 3 (10% FBS + 10% CEE) than in group 2 (20% FBS) at DIV: 7 and 11 (**p < 0.01, one‐way ANOVA followed by post hoc Tukey's test). (D) Also, cell count after the first subculture in experimental group 3 was significantly higher compared to the two other groups (**p < 0.01, one‐way ANOVA followed by post hoc Tukey's test). (E) Assessment of cell proliferation using MTT assay at passage 3 in the different experimental groups showed, that growth medium supplemented with 10% FBS + 10% CEE increased significantly the proliferation rate of stem cells (***p < 0.001, one‐way ANOVA followed by post hoc Tukey's test). (F, G) Comparison of the colony‐forming unit efficiency of hair follicle stem cells treated with different growth media demonstrated that treatment with 10% FBS + 10% CEE generated a significantly larger number of colonies over the culture period. In addition, the number of colonies with 2, 3 and 4 mm in diameter was significantly higher in 10% FBS + 10% CEE treated cell cultures (***p < 0.001; *p < 0.05, two‐way ANOVA followed by post hoc Tukey's test).

Following the treatment of explanted bulges with different growth media, hair bulges were evaluated at DIV 3, 5, 7 and 11 (Figure 2A). Captured images from the same bulges at different days of culture revealed the presence of migrating stem cells at DIV 3 in all three experimental groups with increasing cell numbers over culture time (Figure 2B). However, the percentage of bulges with migrating stem cells was higher in experimental group 3, which was supplemented with 10% FBS + 10% CEE and it significantly increased at DIV 7 and 11, compared to experimental group 2 that was supplemented with 20% FBS (Figure 2C). In addition, the number of stem cells after the first subculture was significantly higher than in experimental group 3 than in the other groups (Figure 2D). Further, an MTT assay performed at passage 3 confirmed a higher proliferation rate of stem cells grown in medium supplemented with CEE (Figure 2E). Besides the assessment of proliferation rate in hair follicle stem cells by means of cell counting and MTT assay, the clonogenic and proliferation potential of these stem cells were evaluated by a colony‐forming unit (CFU) assay. Ten percent (±1.4) of passage‐3 seeded hair follicle stem cells in the CEE supplemented group were capable of forming colonies, 76% (±5.6) of which were >1 mm in diameter. Also, the 20% FBS experimental group showed a CFU efficiency of 6% (±0.39), and 44.6% (±3.2) of these colonies had a diameter >1 mm. The 10% FBS treated cells were least capable of colony formation, with a CFU efficiency of 3.2% (±0.29), and only 13.2% (±2.6) of the colonies being larger than 1 mm diameter in size. These findings highlight the self‐renewal capacity of migrating stem cells, which can be increased in the presence of CEE (Figure 2F,G).

### 3.3 CEE supplement preservers the multipotency of hair follicle stem cells

**FIGURE 3:**
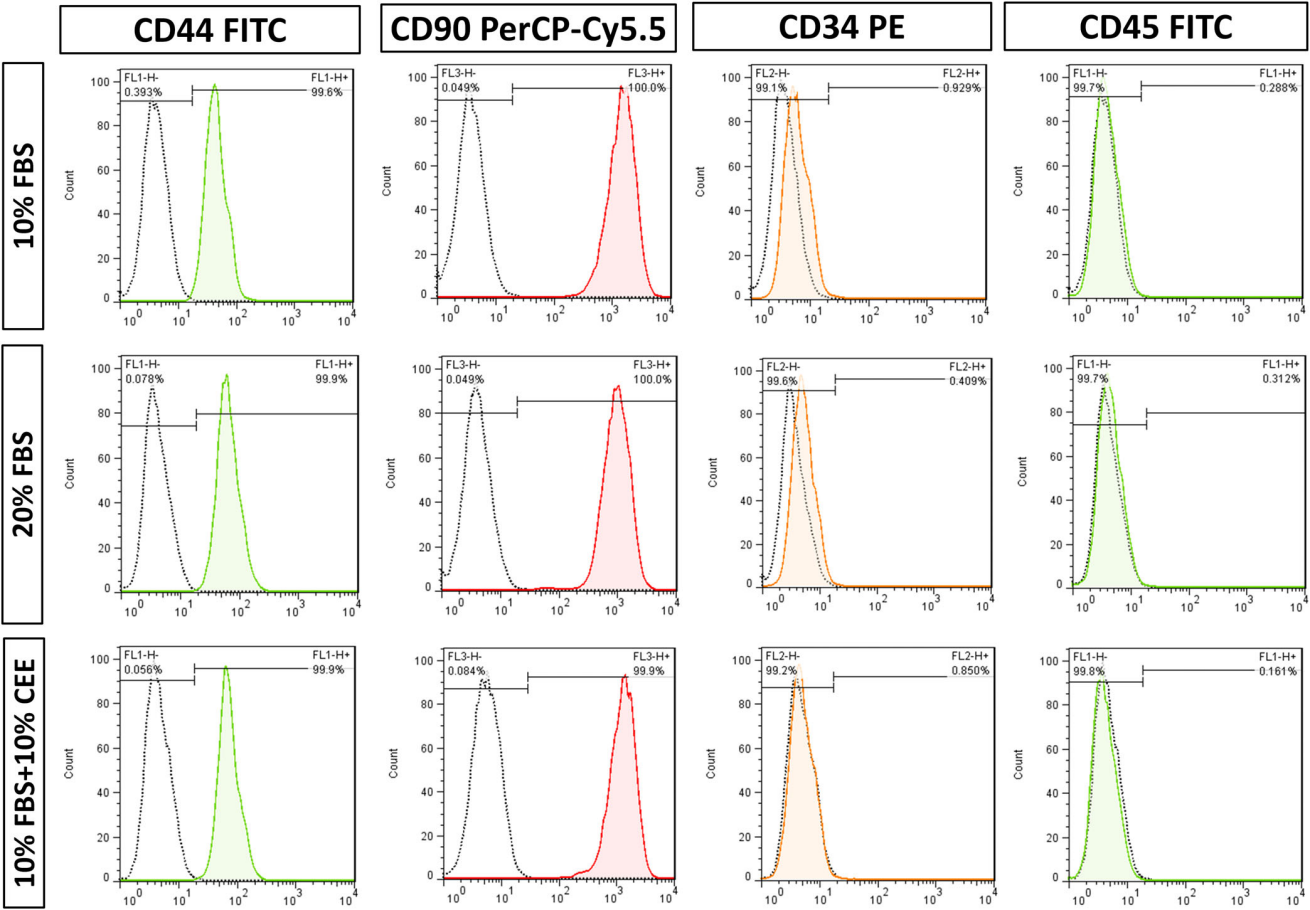
Comparison of multipotency of hair follicle stem cells treated with different growth media. Flow cytometric analysis of key mesenchymal stem cell surface markers revealed, that almost all cells in the three experimental groups expressed CD44 and CD90, but not haematopoietic markers (CD34 and CD45).

Regarding the cranial origin of isolated NCSCs, these hair follicle‐derived stem cells can generate multiple cranial ectomesenchymal structures.^36^ Hence, evaluation of mesenchymal stem cell markers including CD44 and CD90 was carried out. Flow cytometry analysis of cultured hair follicle stem cells at passage 3 showed that all experimental groups featured a similar pattern of expression for CD44, CD90, CD34 and CD45. The frequency of CD44 positivity was more than 99.6% in all three groups. In addition, nearly all counted cells expressed cell‐surface marker CD90, while they were negative for CD34 and CD45, which are known markers of haematopoietic stem cells (Figure 3).

**FIGURE 4:**
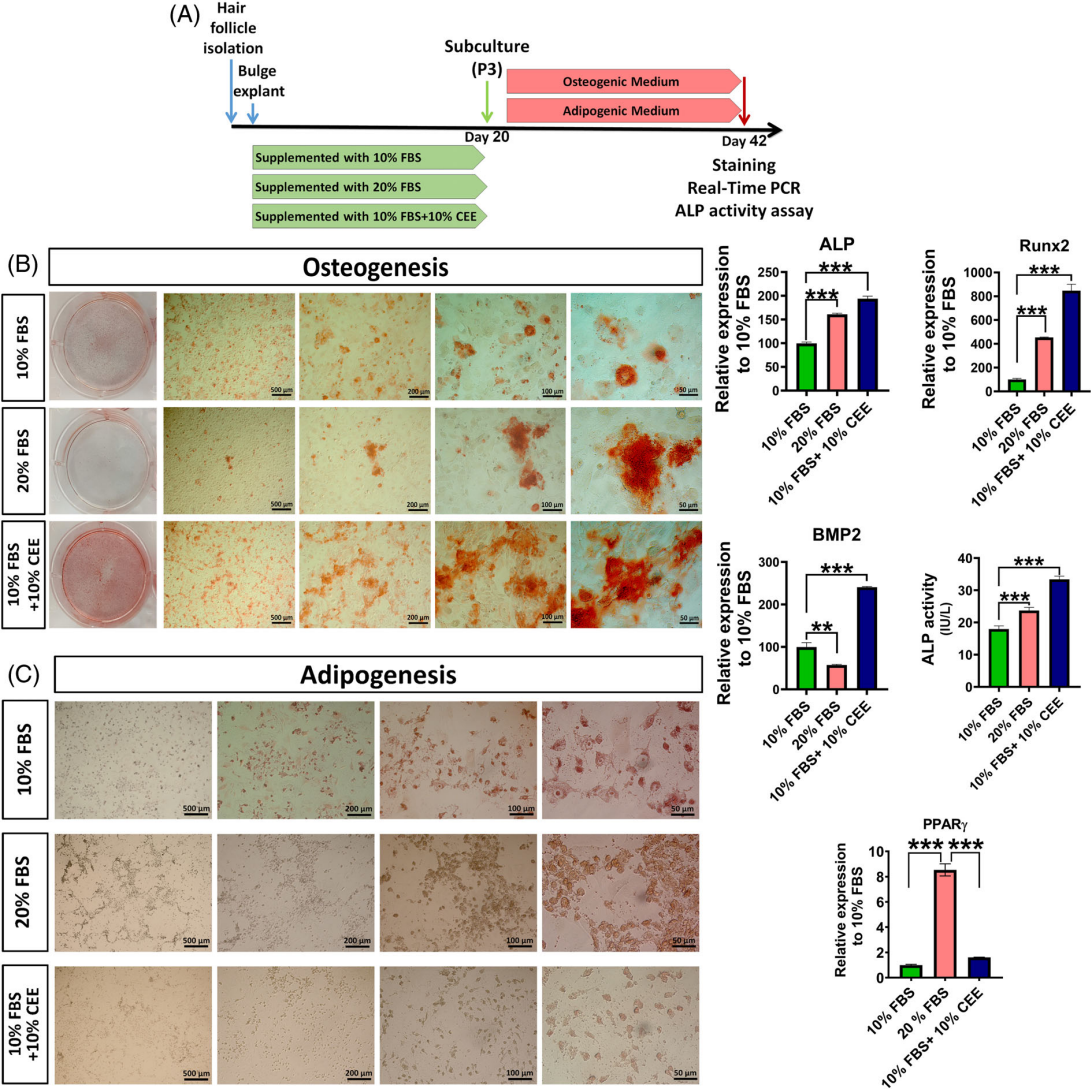
Comparison of osteogenic and adipogenic potential of hair follicle stem cells preconditioned with different growth media. (A) Following third subculture, cells of all experimental groups were seeded in 6‐well plates and incubated with induction medium for 21 days. (B) Alizarine Red staining of cells after 21 days in osteogenic medium showed increased bone matrix mineralization in the chick embryo extract (CEE)‐supplemented group. The density of calcification in the 10% fetal bovine serum (FBS) supplemented group was lower than in the CEE treated group, and the presence of calcified nodules was reduced in the 20% FBS supplemented cells. Evaluation of osteogenic markers of ALP, Runx2 and BMP2 showed increased expression of these genes in CEE supplemented group and higher activity of ALP enzyme was detected in this group, as well. (C) Oil Red O staining reveals an increase in the formation of lipid‐droplets in cells supplemented with 20% FBS. In contrast, cells that contained lipid vesicles were rare in cultures treated with 10% FBS + 10% CEE and 10% FBS only. Also, PPAR‐γ transcription was downregulated in 10% FBS + 10% CEE and in 10% FBS treated cells, respectively (***p < 0.001, **p < 0.01, one‐way ANOVA followed by post hoc Tukey's test).

To investigate the nature and capacity of hair follicle stem cells for osteogenic and adipogenic differentiation, differentiation assays were performed (Figure 4A). Hair follicle stem cells of the different experimental groups cultured under osteogenic conditions developed different osteoblastic phenotypes. Alizarin red staining demonstrated the presence of abundant calcified nodules in cultures of the 10% FBS + 10% CEE treated group. This type of nodules was sparse in cells cultured in medium supplemented with 20% FBS. Moreover, the density of calcification in cells grown in 10% FBS supplemented medium was lower than in the 10% FBS + 10% CEE group. Interestingly, evaluation of transcript level of some osteogenic markers including ALP, runt‐related transcription factor 2 (Runx2) and bone morphogenic protein 2 (BMP2) revealed elevated expression of these genes in CEE supplemented group grown in osteogenic medium. Consistent with these histological and gene expression findings, an enhanced activity of ALP enzyme was detected in CEE group (Figure 4B). These results suggest, that CEE preconditioning markedly induced osteoblast differentiation of hair follicle stem cells. This finding is in line with previous reports showing that CEE preconditioning supports osteogenic differentiation of bone marrow mesenchymal stem cells in ageing rats.^41^

Cultivation of hair follicle stem cells under adipogenic conditions stimulated their differentiation into adipocytes, as assessed by Oil Red O staining. Figure 4C shows that treatment with 20% FBS increased the accumulation of lipid‐filled cells as evidenced by the greater number and size of droplets per cell. Notably, Oil Red O staining in the 10% FBS + 10% CEE group showed fewer reddish‐brown droplets per microscopy field than in the 20% FBS group. Here, the number of stained lipid droplets in the 10% FBS supplemented group was less than in the two other experimental groups. To further compare the propensity for adipogenic differentiation between experimental groups, the transcription levels of peroxisome proliferator‐activated receptor‐gamma (PPAR‐γ) was analysed by qRT‐PCR. This data reveals a decrease in the transcript level of PPAR‐γ in 10% FBS + 10% CEE and 10% FBS treated cells, compared to 20% FBS supplemented cells (Figure 4C). This evidence verifies the multipotency of migrated stem cells under CEE supplementation.

### 3.4 Morphological alterations following CEE treatment

**FIGURE 5:**
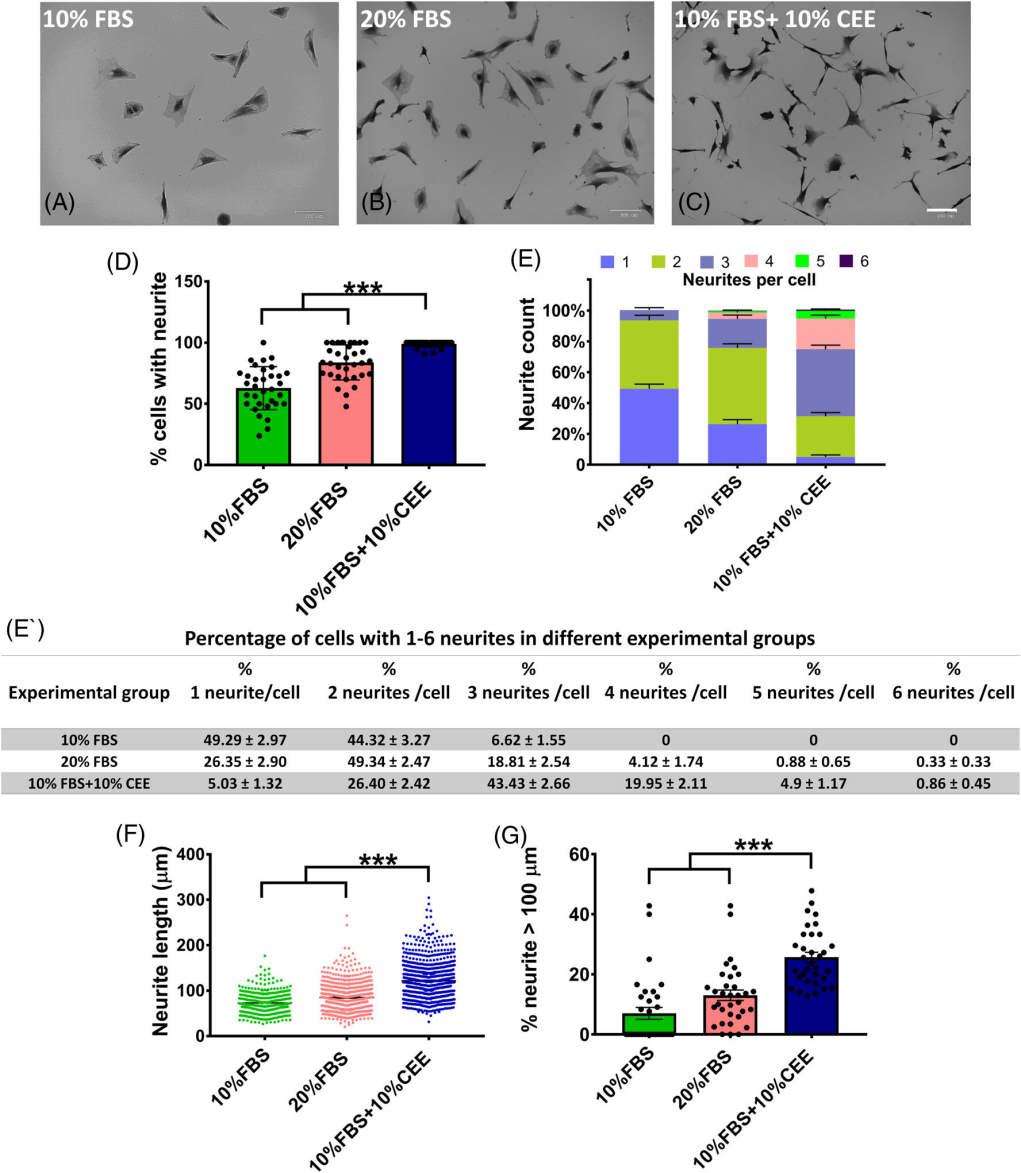
Morphological evaluation of stem cells in different experimental groups. (A–C) The representative images of the experimental groups taken 12 h after the third passage depict morphological differences between them, scale bar: 100 μm. (D) In this study, the chick embryo extract (CEE) supplemented experimental group yielded a higher percentage of cells with neurites. (E, E′) Of the neurite growing cells in the 10% fetal bovine serum (FBS) group, the majority grew only one or two neurites, but not more than three. In the 20% FBS group, roughly 5% of cells grew more than three neurites, while in the CEE supplemented group cells growing 3 and more neurites dominated. (F) Measurement of neurite length showed that cells in CEE group had significantly longer neurites compared to all other groups. (G) Also, the percentage of cells with neurites longer than 100 μm in this group was significantly greater than in the other two groups (***p < 0.001, one‐way ANOVA followed by post hoc Tukey's test).

The morphological assessment of stem cells after treatment with the respective growth media revealed morphological differences between experimental groups (Figure 5A–C). Here, the 10% FBS supplement induced neurite outgrowth in only 62% of the cells. In contrast, 99% of stem cells cultivated in medium supplemented with 10% FBS + 10% CEE showed neurite outgrowth (Figure 5D). While 20% FBS seemed almost as efficient as 10% FBS + 10% CEE in inducing neurite outgrowth in EPI‐NCSCs (83%), this may not hold true for functionality. Neurite functionality is determined by neurite count per cell and length,^42^, ^43^ which allows neurons to connect via synaptogenesis and interact with each other. Indeed, 10% FBS + 10% CEE induced a significantly higher number of neurites per cell (Figure 5E,E′), and those neurites were significantly longer (Figure 5F). In detail, 25.71% of cells treated with 10% FBS + 10% CEE had neurites longer than 100 microns (Figure 5G).

### 3.5 CEE induces spheroid formation in EPI‐NCSCs

**FIGURE 6:**
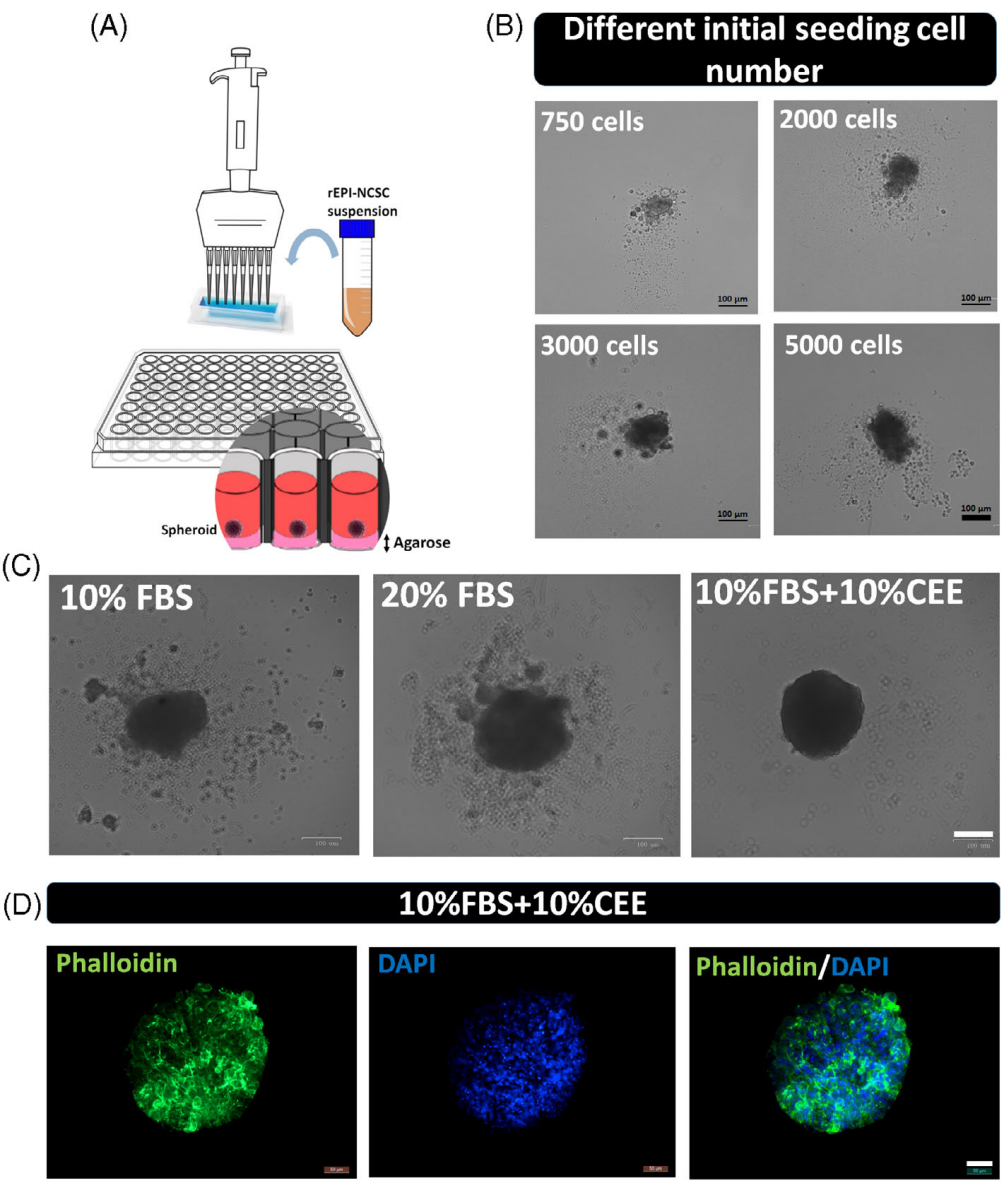
Evaluation of spheroid formation in different experimental groups. (A, B) To define the ability of stem cells to form spheroids on agarose coated 96‐well plates, different numbers of cells were seeded, with 5000 cells per well yielding an appropriate spheroid morphology, scale bar: 100 μm. (C) Next, employing the same initial seeding cell number of 5000 in each of the experimental groups, cells in group 3 (supplemented with 10% fetal bovine serum + 10% chick embryo extract) were found to form a single tight round neurosphere with a minimum of unattached cells around the main core, scale bar: 100 μm. (D) Also, immunostaining against phalloidin and DAPI revealed the shape of spheroid in experimental group 3 by staining of cytoskeletal filaments and nuclei, scale bar: 50 μm.

Growth medium composition also affected the ability of hair follicle stem cells to form spheroids in vitro. To define the capacity of stem cells to create spheroids on agarose‐coated 96‐well plates (Figure 6A), initial seeding cell numbers of 750, 2000, 3000 and 5000 were selected. According to the captured images, 5000 cells were needed to form tight spheroids (Figure 6B). Next, the same initial cell number of 5000 was seeded for each of the three experimental groups. Cells in experimental group 3 (supplemented with 10% FBS + 10% CEE) formed single tight round neurospheres with a minimum of unattached cells around the main core (Figure 6C), while in other experimental groups there were more than one cell aggregate (data not shown) with a significant number of unattached cells remaining. Immunostaining with phalloidin and DAPI revealed the shape of spheroid in experimental group 3 by staining of cytoskeletal filaments and nuclei, respectively (Figure 6D).

### 3.6 CEE alters the gene expression profile of EPI‐NCSCs

**FIGURE 7:**
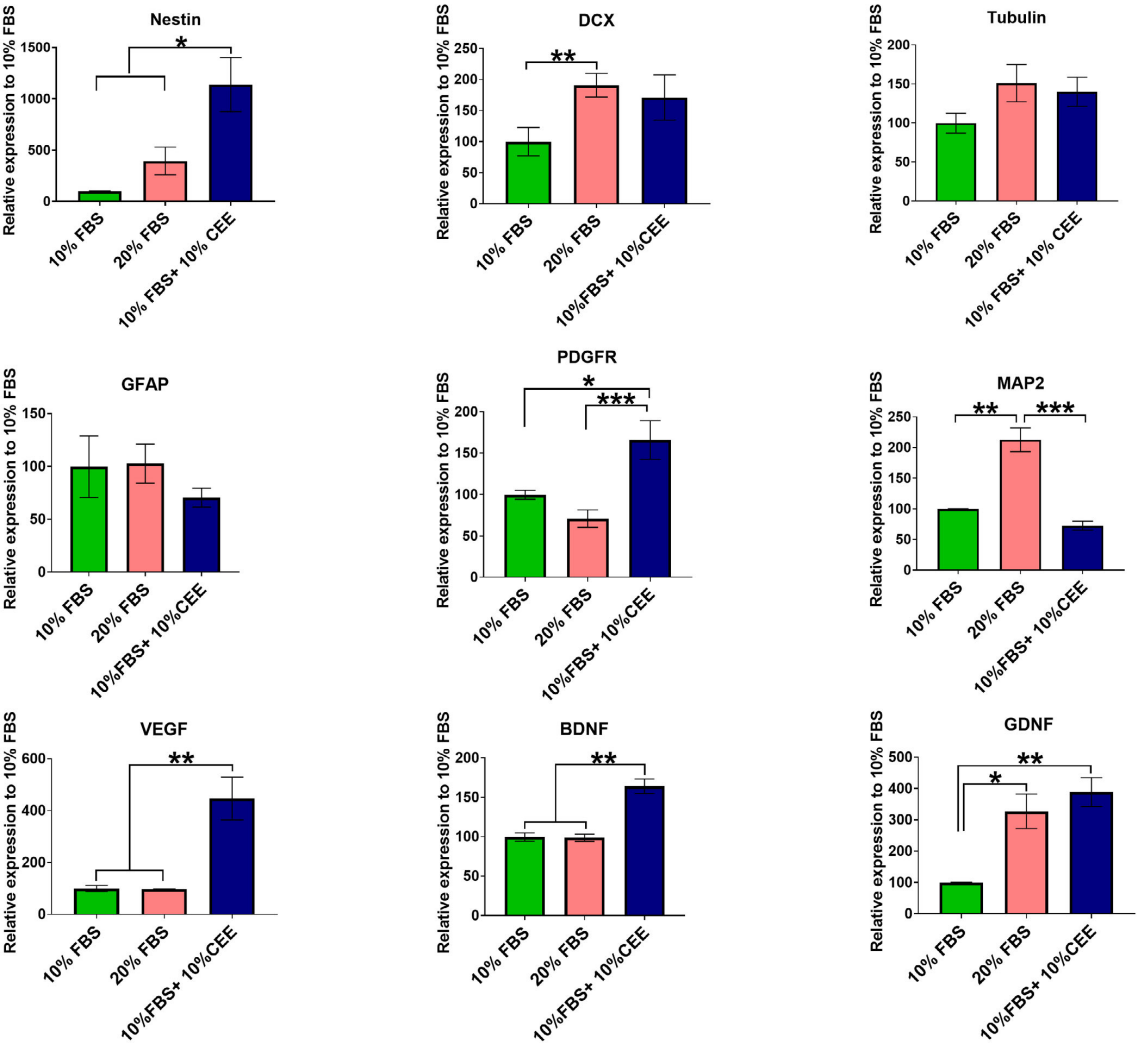
The gene expression pattern of hair follicle stem cells. The gene expression level of Nestin increased significantly in chick embryo extract (CEE) treated stem cells. At the same time, cells grown in 20% fetal bovine serum (FBS) supplemented medium expressed higher level of doublecortin transcript. Also, expression level of neuronal (β‐III Tubulin) and glial‐lineages markers (GFAP and PDGFR‐α) remained unchanged in all three experimental groups, while MAP2 expression (neuron‐specific cytoskeletal marker) significantly increased in the 20% FBS group. Interestingly, CEE treatment induced expression of VEGF, BDNF and GDNF in hair follicle stem cells. The expression of target genes was normalized against the housekeeping gene HPRT. Values are mean ± SEM of three independent experiments and one‐way ANOVA and Tukey's post hoc tests were performed to test for statistical differences among the means. *p < 0.05; **p < 0.01; ***p < 0.001

To obtain further insight into the molecular features of hair follicle stem cells under different culture conditions, we evaluated the mRNA expression levels of key genes connected to stemness, cellular markers of early neural lineages and growth factors (Figure 7). The gene expression level of the NCSC marker nestin increased significantly in the CEE supplemented group, indicating the ability of CEE to preserve stem cell character. At the same time, expression of doublecortin (DCX; a neuronal precursor marker) decreased, indicating that the inability of CEE treatment to promote neuronal fate acquisition. In contrast, supplementation with 20% FBS increased significantly the level of DCX transcript. Moreover, the expression level of β‐III tubulin (early neuronal marker), or GFAP (astrocyte marker) remained unchanged, and PDGFR‐α (oligodendrocyte precursor marker) significantly increased in CEE treated stem cells. A significantly elevated mRNA level of MAP2 (microtubule‐associated protein 2), which is a neuron‐specific cytoskeletal marker (enriched in dentrites and perikarya), was specific for the 20% FBS group. The higher expression of DCX and MAP2 in the 20% FBS supplemented group indicates the tendency of these stem cells towards neuronal lineage differentiation. High levels of the growth factors brain‐derived neurotrophic factor (BDNF) and glial cell line‐derived neurotrophic factor (GDNF), and of vascular endothelial growth factor (VEGF) were detected in CEE supplemented stem cells (Figure 7). Elevated growth factors levels may mediate stem cell therapeutic potential and successful tissue regeneration.

### 3.7 CEE affects the cell fate of EPI‐NCSCs

**FIGURE 8:**
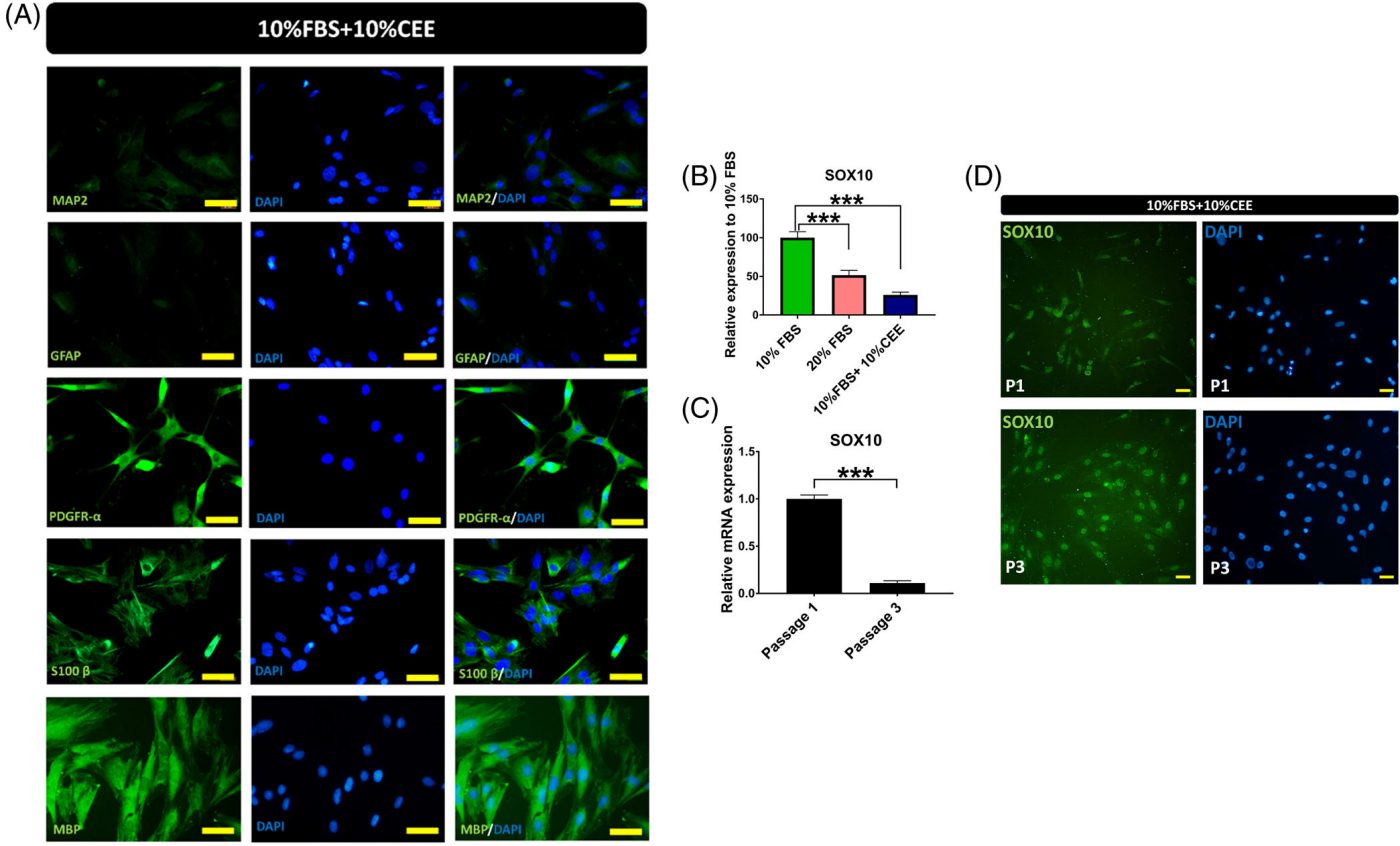
Protein expression of hair follicle stem cells in presence of chick embryo extract (CEE). To define fate of CEE treated stem cells, immunostaining was carried out against six markers. (A) Here, very low fluorescent intensity was detected after MAP2 and GFAP staining, while strong expression of PDGR‐α, S100 B and MBP was observed in these stem cells, Scale bar: 50 μm. (B) Evaluation of SOX10 gene expression by RT‐PCR revealed the significant reduction of SOX10 transcript in the CEE group. (C) Comparison of SOX10 transcript levels between passage 1 and 3 showed a 90% decrease in SOX‐10 expression in passage 3 (***p < 0.001, one‐way ANOVA followed by post hoc Tukey's test). However, strong expression of SOX10 protein was observed in both passages with quite different expression patterns (***p < 0.001, one‐way ANOVA followed by post hoc Tukey's test). (D) In passage 1, SOX10 was equally distributed between the nucleus and cytoplasm or exclusively in the cytoplasm. While in passage 3, this transcription factor localized mainly to the nucleus, Scale bar: 25 μm. The micrographs are representative images of four independent experiments.

Immunostaining was performed to determine the cell fate of CEE treated stem cells. We found that in concordance with gene expression analysis, MAP2 and GFAP proteins were also expressed at very low levels. In contrast, the majority of cells expressed PDGFR‐α, S100 B and MBP (myelin basic protein) at the protein level (Figure 8A). S100 B is a calcium binding protein that is prevalently expressed in glial cells.^44^, ^45^ Also, MBP is one of the major proteins of the CNS that is abundantly expressed in the myelin sheath and is essential for the formation of the dense line. Considering the expression of Nestin (Figure 1F), enhanced levels of PDGFR‐α transcript and protein and expression of S100 B and MBP in this experimental group, it can be hypothesized that CEE content may govern the differentiation of these stem cells towards the glial lineage and more specifically into Schwann cells.

Besides evaluation of the aforementioned genes and proteins, the expression of SOX10 as a key transcriptional regulator of neural crest development was assessed. SOX10 is preferentially and abundantly expressed in glial cells and it is involved in lineage specification.^46^ To define the influence of CEE on SOX10 activity, we evaluated the expression of SOX10 in our three experimental groups. SOX10 expression was significantly decreased in the CEE supplemented group at passage 3 (Figure 8B). The comparison of SOX10 transcript levels between passages 1 (after their first subculture) and 3 by RT‐PCR, revealed that SOX10 transcript levels were approximately 9‐fold higher in passage 1 than passage 3 (Figure 8C). However, in contrast to the qPCR‐results, immunostaining showed persistent SOX10 protein expression in passage 3, mostly in the nucleus, where it binds target promoter and enhancer elements to regulate gene expression. In contrast, SOX10 was equally distributed between nucleus and cytoplasm or exclusively found in the cytoplasm of CEE treated stem cells in passage 1 (Figure 8D).

### 3.8 Proteome content of the CEE

Proteome analysis of CEE by tandem mass spectrometry identified 833 protein groups in a BLAST sequence similarity against the unreviewed UniProtKB/TrEMBL database, and 287 protein groups against the curated and non‐redundant UniProtKB/Swiss‐Prot database. We assigned the identified protein groups by their gene ontology functions to either general transcription factors, stem cell maintenance, or neuronal differentiation/migration/morphology‐related factors (see Tables 2, 3, and 4). In order to identify relevant transcription factors in Table 2, we performed an in silico analysis of the top transcription factor binding sites of our genes of interest that we identified to be significantly regulated by CEE treatment (Table 5) and referenced those with factors in CEE that are known to biochemically interact with the transcription factors. In order to isolate the functionally relevant factors from the CEE mixture, we mapped the promoters of genes that we found to be regulated in EPI‐NCSCs. The transcription factor binding sites within the promoters guide us to scan the CEE proteome for activators of those transcription factors. We identified gamma calcium/calmodulin‐dependent kinase II (γCamKII) in the CEE, which is a known activator of the transcription factor of cyclic AMP response element binding protein (CREB), for example, direct evidence for CamKII‐mediated activation of CREB‐regulated BDNF expression comes from cultured rat postnatal dorsal root ganglion neurons.^47^ CREB not only regulates BDNF, but also GDNF and VEGF transcription. CREB, among other transcription factors, is also a known regulator of neurite outgrowth.^48^

**TABLE 2:** Factors involved in gene expression identified in the CEE by mass spectrometry.

Activated RNA polymerase II transcriptional coactivator p15 (SUB1 homologue)
Transcription factor BTF3
14‐3‐3 protein theta
Acidic leucine‐rich nuclear phosphoprotein 32 family member B
Actin‐related protein 3 (Actin‐like protein 3)
Alpha‐actinin‐4 (Non‐muscle alpha‐actinin 4)
Brain acid soluble protein 1 homologue (23 kDa cortical cytoskeleton‐associated protein) (CAP‐23)
Calcium/calmodulin‐dependent protein kinase type II delta chain (CaM kinase II subunit delta) (CaM‐kinase II delta chain) (CaMK‐II subunit delta) (EC 2.7.11.17)
Eukaryotic translation initiation factor 2 subunit 1 (Eukaryotic translation initiation factor 2 subunit alpha) (eIF‐2‐alpha) (eIF‐2A) (eIF‐2alpha)
Eukaryotic translation initiation factor 2 subunit 3 (EC 3.6.5.3) (Eukaryotic translation initiation factor 2 subunit gamma) (eIF‐2‐gamma)
Eukaryotic translation initiation factor 3 subunit E (eIF3e) (Eukaryotic translation initiation factor 3 subunit 6)
Eukaryotic translation initiation factor 3 subunit H (eIF3h) (Eukaryotic translation initiation factor 3 subunit 3) (eIF‐3‐gamma) (eIF3 p40 subunit)
Eukaryotic translation initiation factor 3 subunit L (eIF3l)
Eukaryotic translation initiation factor 5A‐1 (eIF‐5A‐1) (eIF‐5A1) (Eukaryotic initiation factor 5A isoform 1) (eIF‐5A) (eIF‐4D)
Eukaryotic translation initiation factor 5A‐2 (eIF‐5A‐2) (eIF‐5A2) (Eukaryotic initiation factor 5A isoform 2) (eIF‐4D)
Fatty acid‐binding protein, brain (Brain‐type fatty acid‐binding protein) (B‐FABP) (Fatty acid‐binding protein 7) (Fatty acid‐binding protein, retina) (R‐FABP)
Fatty acid‐binding protein, liver (Fatty acid‐binding protein 1) (Liver basic FABP) (LB‐FABP) (Liver bile acid‐binding protein) (L‐BABP) (Liver‐type fatty acid‐binding protein) (L‐FABP)
Fatty acid‐binding protein, smooth muscle (SM‐FABP) (Fragments)
Glyceraldehyde‐3‐phosphate dehydrogenase (GAPDH) (EC 1.2.1.12) (Peptidyl‐cysteine S‐nitrosylase GAPDH) (EC 2.6.99.‐)
Heterogeneous nuclear ribonucleoprotein K (hnRNP K)
High mobility group protein B1 (High mobility group protein 1) (HMG‐1)
High mobility group protein B2 (High mobility group protein 2) (HMG‐2)
High mobility group protein B3 (High mobility group protein 2a) (HMG‐2a) (High mobility group protein 4) (HMG‐4)
Lumican (Keratan sulfate proteoglycan lumican) (KSPG lumican)
Mothers against decapentaplegic homologue 3 (MAD homologue 3) (Mad3) (Mothers against DPP homologue 3) (SMAD family member 3) (SMAD 3) (Smad3)
Myotrophin (Granule cell differentiation protein) (Protein V‐1)
Nucleolin (Protein C23)
Nucleophosmin (NPM) (Nucleolar phosphoprotein B23) (Nucleolar protein NO38) (Numatrin)
Nucleoside diphosphate kinase (NDK) (NDP kinase) (EC 2.7.4.6)
Paraspeckle component 1 (PSPC1)
Proliferating cell nuclear antigen (PCNA)
Pterin‐4‐alpha‐carbinolamine dehydratase (PHS) (EC 4.2.1.96) (4‐alpha‐hydroxy‐tetrahydropterin dehydratase) (Dimerization cofactor of hepatocyte nuclear factor 1‐alpha) (DCoH) (Dimerization cofactor of HNF1) (cDcoH) (Phenylalanine hydroxylase‐stimulating protein) (Pterin carbinolamine dehydratase) (PCD)
Serine‐threonine kinase receptor‐associated protein
S‐phase kinase‐associated protein 1 (Cyclin‐A/CDK2‐associated protein p19) (S‐phase kinase‐associated protein 1A) (p19A) (p19skp1)
Spliceosome RNA helicase DDX39B (EC 3.6.4.13) (56 kDa U2AF65‐associated protein) (DEAD box protein UAP56)
Thioredoxin (Trx) stimulates AP‐1 activity
Translationally controlled tumour protein homologue (TCTP) (p23) (pCHK23)
Ubiquitin carboxyl‐terminal hydrolase 7 (EC 3.4.19.12) (Deubiquitinating enzyme 7) (Ubiquitin thioesterase 7) (Ubiquitin‐specific‐processing protease 7)
Ubiquitin‐40 S ribosomal protein S27a (Ubiquitin carboxyl extension protein 80) [Cleaved into: Ubiquitin; 40 S ribosomal protein S27a]
Ubiquitin‐conjugating enzyme E2 variant 1 (UEV‐1) (CROC‐1B)
Y‐box‐binding protein 1 (YB‐1) (Y‐box transcription factor)

**TABLE 3:** Stem cell maintenance‐related factors identified in the CEE by mass spectrometry.

Acidic leucine‐rich nuclear phosphoprotein 32 family member B
Acidic leucine‐rich nuclear phosphoprotein 32 family member E
Eukaryotic translation initiation factor 5A‐1 (eIF‐5A‐1) (eIF‐5A1) (Eukaryotic initiation factor 5A isoform 1) (eIF‐5A) (eIF‐4D)
Eukaryotic translation initiation factor 5A‐2 (eIF‐5A‐2) (eIF‐5A2) (Eukaryotic initiation factor 5A isoform 2) (eIF‐4D)
Probable ATP‐dependent RNA helicase DDX6 (EC 3.6.4.13) (DEAD box protein 6)
Translationally controlled tumour protein homologue (TCTP) (p23) (pCHK23)

**TABLE 4:** Neuronal differentiation, migration and morphology‐related factors identified in the CEE by mass spectrometry.

Basic leucine zipper and W2 domain‐containing protein 2
Calbindin (Calbindin D28) (D‐28K) (Vitamin D‐dependent calcium‐binding protein, avian‐type)
Dihydropyrimidinase‐related protein 2 (DRP‐2) (Collapsin response mediator protein CRMP‐62)
Twinfilin‐2
Tubulin beta‐1 chain (Beta‐tubulin class‐I)
UDP‐glucose 6‐dehydrogenase (UDP‐Glc dehydrogenase) (UDP‐GlcDH) (UDPGDH) (EC 1.1.1.22)

**TABLE 5:** Transcription factor binding sites within the promoter of CEE‐regulated target genes (CEE TF)

CEE TF	TF‐binding sites in promoter region								
Nestin	*AhR*	*Bach1*	*E47*	*IK‐3*	*OLF‐1*	*Pax‐5*	*RP58*	*Tal‐1ß*	*c‐Myc*
PDGFR	*AML1a*	*c‐Myb*	*c‐Myc*	*c‐Rel*	*COMP1*	*GATA‐1*	*Max1*	*SRY*	*YY1*
VEGF	*PPAR‐α*	*PPAγ‐1*	*CREB*	*c‐Myc*					
BDNF	*NRSF*	*CREB*	*USF1/2*	*CaRF*					
GDNF	*CREB*	*δ‐CREB*							
MAP2	*AP‐1*	*C/EBPα*	*MyoD*						
Sox‐10	*AP‐2γ*	*AREB6*	*E47*	*HOXA5*	*LMO2*	*NF‐1*	*NF1/L*	*RREB‐1*	*USF‐1*
DCX	*AP‐1*								
S100B	*AP‐1*	*AP‐2α* (*2*,*3*,*4*,*A*)	*PPAR‐γ1*	*PPAR‐γ2*					
MBP	*AP‐1*	*PPAR‐α*							

*Note*: AP‐1: AP‐1 activity regulates Sox‐10, S100B and MBP expression and is induced by Thioredoxin (see Table 2). BDNF, GDNF, as well as VEGF expression is regulated by the transcription factor CREB (Jeon et al., 2007), which in turn is activated by γCamKII (see Table 2). Nestin, PDGFR and VEGF expression are regulated by the transcription factor c‐Myc, which is under the control of the signalling factor SMAD3 (see Table 2).

Another potentially important factor in the CEE might be thioredoxin, which is a critical activator of the transcription factor activating protein‐1 (AP‐1). AP‐1 has been shown to regulate cell proliferation and migration as well.^49^ AP‐1 is composed of several subunits of the Jun and Fos family transcription factors. Thioredoxin‐mediated activation of these factors may drive the transcription of SOX‐10, S100B and MBP.^50^ Other AP‐1 activators might also be effective; however, the presence of thioredoxin in the CEE and the activation of AP‐1‐dependent genes is certainly striking. In order to effectively activate a transcription factor, thioredoxin has to be incorporated into the target cell and released intracellularly. It has been shown that thioredoxin is released into the extracellular space via exosomes, and can therefore, also be taken up by target cells via specific receptors or the endocytotic pathway.^51^ Exosomes are membrane‐bound extracellular vesicles that are produced in the endosomal compartment of most eukaryotic cells with roughly 30–150 nm in size, which contain proteins, RNA and lipids, rendering exosomes extremely useful for therapeutic regimens for the treatment of cancer, pregnancy complications, infections, or autoimmune diseases.^51^

Another AP‐1 dependent factor is the translationally controlled tumour protein (TCTP), also called histamine releasing factor or fortilin. TCTP is a multifunctional protein expressed abundantly in eukaryotic organisms. TCTP binds to and stabilizes microtubules, and thereby orchestrates a range of basic cell biological processes, such as cell growth, proliferation, cytoskeletal organization/cellular morphology and decreased directionality during migration.^52^, ^53^ Mechanistically, TCTP acts as an anti‐apoptotic protein and it is involved in DNA‐damage repair.^54^ Apart from the above mentioned processes, TCTP is a critical protein for cell survival during early neuronal and glial differentiation.^55^ This protein as a key regulatory molecule is involved in expanding the neural precursor pool. TCTP is among the most abundant transcripts in adult axons^56^ which its elevated level has been reported after sciatic nerve injury^57^ and spinal cord injury.^58^ Intriguingly, TCTP can also be released into the extracellular space via exosomes^59^ and re‐enter our target cells in a similar way like thioredoxin. A third candidate for an extracellular mitogenic and pro‐migratory factor is Y‐box‐binding protein 1 (YB‐1). YB‐1 inhibits apoptosis but promotes proliferation, migration, invasion and angiogenesis. YB‐1 is a transcription co‐factor,^60^ but can also be secreted and acts as an extracellular mitogen.^61^ Secretion is not mediated by classical exosomic pathways, but rather in a non‐classical way via microvesicles and ATP‐binding cassette transporters.^61^ Furthermore, Nestin, PDGFR and VEGF contain a c‐Myc binding sequence within their promoters, indicating an important role for c‐Myc in the activation of gene expression by the CEE. C‐Myc is activated by a variety of different factors, like IGF‐1, TGFα, Interleukin‐6, Wnt signalling or the TGFβ pathway. The TGFβ pathway contains mothers against decapentaplegic homologue 3 (SMAD3) proteins, which activate nuclear c‐Myc, and therefore likely drive Nestin, PDGFR, and VEGF expression. SMADs also form physical complexes with AP‐1 to drive gene transcription, which would also affect the regulation of the above‐mentioned AP‐1‐dependent genes.^62^ Moreover, exosome recognition via the TGFß pathway has been shown to activate c‐Myc,^63^ providing a secondary mechanism how thioredoxin‐, or fortilin‐containing exosomes can activate mitogenic, migratory, angiogenic, or morphological factors. However, we like to emphasize that gene expression is a multi‐factorial process, with dozens of different transcription factors tightly regulating the expression of one single gene. Therefore, our findings on CEE‐borne factors that are able to bind the promoters of our target genes do not claim to be comprehensive but provide a first insight into a set of essential factors that are present in the CEE.

## 4 DISCUSSION

The role of EPI‐NCSCs, isolated from different animal species, has been extensively studied over the last two decades.^64^ In particular, these stem cells have been reported to exert beneficial effects in animal models of spinal cord injury,^16^, ^65^, ^66^, ^67^ sciatic nerve injury,^21^, ^68^, ^69^ and brain ischemic stroke.^22^ CEE has been used as a routine growth medium supplement for the cultivation of hair follicle stem cells. To the best of our knowledge, this is the first study to reveal a substantial effect of CEE on EPI‐NCSCs. We demonstrated that CEE significantly enhanced the migration rate of stem cells out of hair bulges and induced their proliferation in vitro. Regarding the isolation of hair follicles from the cranial region, stem cells in all three experimental groups expressed mesenchymal stem cell surface markers and preserved their capability to differentiate into both, osteogenic and adipogenic lineages. However, CEE supplementation obviously directed the fate of these cells towards the osteogenic lineage following induction in osteogenic medium. The molecular data revealed that several CEE‐derived growth factors can alter the gene and protein expression profiles of hair follicle stem cells. Growth medium containing CEE, substantially affected the morphology of EPI‐NCSCs. Our results show that cells grown in 10% FBS and 10% CEE exhibit neural morphological features as evidenced by the presence of 3–5 neurites of substantial length per cell. Moreover, CEE significantly enhanced their ability to form spheroids.

Despite the hitherto poor characterization of trophic components present in CEE, this supplement has been widely used for the cultivation of different cell types including NCSCs, neuroepithelial cells,^70^, ^71^ stem cells isolated from skeletal muscle^72^ and glomerular cells.^73^ Previously, Maxwell et al.^74^ reported the pivotal role of CEE in combination with synthetic GDNF in promoting the development of adrenergic neurons in mouse neural crest cultures. Furthermore, the crucial role of CEE was demonstrated in the survival and neural crest marker expression of crestospheres, which are in vitro maintained primary cultures of premigratory neural crest cells.^75^ The culture medium containing CEE allows long‐term maintenance of multipotent cranial‐^76^ and trunk‐derived crestospheres^77^ in a premigratory stem or early progenitor state in vitro that are valuable resources for probing mechanisms underlying neural crest stemness and lineage decisions as well as related diseases.

In this study, supplementation of cell culture medium with CEE increased the percentage of bulges with migrating stem cells to almost 90%. This subject is critical in the cultivation of human hair bulges, as only few of bulges can be obtained from human skin punches for explantation. Based on our observations, the supplementation of CEE significantly increased stem cell proliferation, as shown by cell counting, MTT and CFU assay. This finding significantly elevates the potential of these stem cells for cell‐based therapies as it shortens the time period required to obtain a sufficient number of stem cells with clonogenic properties for cell‐based therapies. This data is in line with previous studies, which reported that the increased growth rate of a spontaneously immortalized chicken embryo fibroblast cell line was achieved with CEE treatment.^78^ Also, Bałaban and colleagues used CEE to enhance the proliferation of muscle progenitor cells^79^ and Ma et al. revealed its positive effect on the proliferation rate of bone marrow‐derived mesenchymal stem cells of ageing rats.^41^

In addition to the successful preclinical application of hair follicle stem cells, isolated from different animal species, in various neurological conditions, human hair follicle stem cells are attractive candidates for disease modelling, drug discovery and cell‐based therapies.^80^, ^81^ Two essential parameters that need to be considered before human hair follicle stem cell transplantations become possible are the limitations of scarce skin biopsies and the problem of delivering adequate numbers of stem cells. CEE is a potential cell culture adjuvant that is able to enhance both migration and proliferation rate of hair follicle stem cells, which might help to overcome those limitations. In addition, our research team succeeded to obtain large‐scale expansions of human hair follicle stem cells from pubic hairy skin of a brain‐death patient who donated his skin (unpublished data). Although previous studies mainly supplemented growth medium with a combination of several growth factors,^82^ in our experiment 10% FBS + 10% CEE yielded a large‐scale expansion of stem cells. According to the proteome content of CEE, the activation of AP‐1 and TCTP in presence of thioredoxin in the CEE might lead to an increased rate of migration and proliferation of stem cells.

In addition to the changes observed in migration and proliferation, CEE addition resulted in alterations of the gene expression profile. CEE contains numerous components that have stimulatory and/or inhibitory roles in stem cell differentiation and cell cycle. Our real time‐PCR data obtained from the experimental group supplemented with CEE revealed an upregulation of VEGF, BDNF and GDNF. Hair follicle stem cells express numerous neurotrophic and angiogenic factors^16^ with VEGF and BDNF being among them. VEGF signalling guides neuronal migration and axon pathfinding independently from its vascular effects.^83^ Besides the neuroprotective effects of VEGF in neurodegenerative disease, it has several contextual roles in various neurological diseases, including trauma, stroke and multiple sclerosis.^84^ BDNF expression was also increased in these stem cells by CEE treatment. BDNF is the most common neurotrophin in the brain and its expression is affected in several neurological conditions.^85^, ^86^ To date, various strategies based on BDNF administration are designed to restore BDNF function in neurodegenerative diseases.^87^ One of the possible therapeutic strategies to increase BDNF levels in the brain is to graft cells that are engineered to stably express this trophic factor.^88^ Expression of BDNF has been confirmed in naïve hair follicle stem cells.^16^ Thus, CEE treatment might serve as a preconditioning strategy to enhance the baseline expression of BDNF, which makes them a proper cell type for transplantation under conditions like ischemic stroke. Another trophic factor whose expression was elevated by CEE treatment was GDNF. The therapeutic potential of GDNF has been extensively studied in different disorders with disturbed dopamine homeostasis^89^ and this treatment strategy might benefit a wide range of psychiatric and neurological disorders.

The expression of a specific set of markers, including Nestin, PDGFR‐α, S100B and MBP, indicate that CEE treatment affects the cell fate of hair follicle stem cells. Given the expression of Nestin protein, increased levels of PDGFR‐α transcript and expression of PDGFR‐α, S100B and MBP protein, it is possible that CEE directs the fate of these stem cells towards the glial lineage, and more specifically Schwann cells. In addition to activation of aforementioned genes and proteins, CEE content alters SOX10 expression. SOX10 functions as a lineage determinant in both glial cell types, and it is a major regulator of differentiation and myelination.^90^ Despite discrepancies on the mRNA level, elevated protein levels and nuclear localization of SOX‐10 protein indicate CEE‐induced activity of this protein. These findings are consistent with two other investigations demonstrated the potential of EPI‐NCSCs to differentiate into Schwann cell in vitro. In 2015, Sakaue and Siber‐Blum reported human Schwann cells could be derived rapidly and in a straightforward way from human EPI‐NCSCs through manipulation of several signalling pathways and exposure of the cells to pertinent growth factors.^12^ More recently, Khodabakhsh and colleagues (2021) suggested the application of insulin to promote Schwann‐like cell differentiation of rat hair follicle stem cells.^91^

Remarkably, morphological alterations of migrated stem cells have occurred following CEE treatment, as cells displayed an increased number and length of branches. This morphometric alteration is the consequence of reorganization of the actin cytoskeleton that probably occurs following a genomic response to the CEE components. The suggested mechanism potentially involves CREB phosphorylation triggered by active CamKII contained in the CEE. Apart from the presence of CamKII in CEE, the TCTP can also interact with the cytoskeleton mostly with actin,^92^ and regulate the cytoskeleton organization and through this action influences cell shape. Another absorbing result of this study was the influence of CEE on the spheroid formation ability of hair follicle stem cells. It has been well documented that hair follicle stem cells due to their stemness property can form spheroids when supplemented with growth factors.^8^, ^93^ Among different emerging skin 3D models,^94^, ^95^ spheroids are a promising platform for simulating the complex cellular structure of the nervous system to study the underlying disease mechanisms.^96^ According to the above‐mentioned components of CEE, preconditioning of hair follicle stem cells with this cocktail can rapidly direct the fate of these stem cells towards Schwann cells that support peripheral nerves. The capacity of the peripheral nerve for regeneration after injury is highly dependent on Schwann cells.^97^ Under pathological conditions like peripheral nerve injury, Schwann cells undergo epigenomic remodelling to de‐differentiate into a repair state. Following this transition, repair Schwann cells contribute to nerve regeneration. Hence, grafting an appropriate source of Schwann cells for therapeutic applications is a major challenge. It has been suggested by Chen and colleagues that Schwann cells in injured peripheral nerves, produce IL‐1β, which in turns promote c‐JUN expression and activation of AP‐1 that participate in the de‐differentiation process and activation of the repair state.^98^ Recently, Ramesh and colleagues reported that c‐JUN upregulates Sonic hedgehog expression, which is a unique marker of repair state in the Schwann cell lineage.^99^ Thus, c‐JUN and AP‐1 play a pivotal role in the regeneration of peripheral nerves. Regarding the presence of Thioredoxin in CEE, which activates c‐JUN and AP‐1, we hypothesized that preconditioning of hair follicle stem cells with CEE might result in the generation of a population of Schwann cells that can significantly support the regeneration process of peripheral nerve injury. Indeed, successful cell transplantation in neurological conditions depends on survival, migration, and proliferation of grafted cells. TCTP is another key element that can be regulated by AP‐1. The capacity of this protein to modulate cellular stress, such as oxidative stress, imbalance of ion metabolism that are main hallmarks of injury site and its antiapoptotic effect, prompt speculation that CEE preconditioning might improve transplantation success of hair follicle stem cells in injury sites.

## 5 CONCLUSION

Collectively, hair follicle stem cells are multipotent adult stem cells that present a valuable resource for nervous system cell‐based therapies. Our findings demonstrate that CEE contains a complex cocktail of trophic factors and cytokines that help hair follicle stem cells to acquire some desirable traits that can enhance their therapeutic benefit without the need for genetic manipulation. Given the increased level of neurotrophins, the angiogenic factor VEGF, and the expression of PDGFR‐α, S100B, MBP and SOX‐10 protein, it is tempting to speculate that CEE treatment directs the fate of these stem cells towards the glial lineage, and more specifically towards Schwann cells. In addition to our findings on CEE‐directed cell fate, we hope to prompt further research to narrow down the effective factors in the CEE cocktail to allow the recombinant synthesis of an animal‐free extract with similar capacities.

## AUTHOR CONTRIBUTIONS

Sareh Pandamooz designed the experiments, conducted the experiments, analysed data and wrote the paper. Benjamin Jurek conducted the experiments and wrote the paper. Mehdi Dianatpour analysed data and wrote the paper. Katharina Limm conducted the experiments. Peter J. Oefner critically revised the manuscript. Silke Haerteis critically revised the manuscript. Leila Dargahi critically revised the manuscript. Jaleel A. Miyan critically revised the manuscript. Afshin Borhani‐Haghighi analysed data and wrote the paper. Mohammad Saied Salehi designed the experiments, conducted the experiments and analysed data. All authors read and approved the final manuscript.

## FUNDING INFORMATION

This research was financially supported by Shiraz University of Medical Sciences (Grant No: 1400‐7879 and 26661).

## CONFLICT OF INTEREST

The authors declare that they have no competing interests.

## Data Availability

The authors declare that all data supporting the findings of this study are available within the article or are available from the corresponding author upon request.

## References

[1] Jarrige M , Frank E , Herardot E , et al. The future of regenerative medicine: cell therapy using pluripotent stem cells and acellular therapies based on extracellular vesicles. Cell. 2021;10(2):240. 10.3390/cells10020240 PMC7912181 33513719

[2] Sivandzade F , Cucullo L . Regenerative stem cell therapy for neurodegenerative diseases: an overview. Int J Mol Sci. 2021;22(4):2153. 33671500 10.3390/ijms22042153 PMC7926761

[3] Perera SN , Kerosuo L . On the road again: establishment and maintenance of stemness in the neural crest from embryo to adulthood. Stem Cells. 2021;39(1):7‐25. 33017496 10.1002/stem.3283 PMC7821161

[4] Chen C‐L , Huang W‐Y , Wang EHC , Tai K‐Y , Lin S‐J . Functional complexity of hair follicle stem cell niche and therapeutic targeting of niche dysfunction for hair regeneration. J Biomed Sci. 2020;27(1):1‐11. 32171310 10.1186/s12929-020-0624-8 PMC7073016

[5] Sieber‐Blum M , Hu Y . Epidermal neural crest stem cells (EPI‐NCSC) and pluripotency. Stem Cell Rev. 2008;4(4):256‐260. 18712509 10.1007/s12015-008-9042-0

[6] Amoh Y , Kanoh M , Niiyama S , et al. Human and mouse hair follicles contain both multipotent and monopotent stem cells. Cell Cycle. 2009;8(1):176‐177. 19106614 10.4161/cc.8.1.7342

[7] Narytnyk A , Verdon B , Loughney A , et al. Differentiation of human epidermal neural crest stem cells (hEPI‐NCSC) into virtually homogenous populations of dopaminergic neurons. Stem Cell Rev Rep. 2014;10(2):316‐326. 24399192 10.1007/s12015-013-9493-9 PMC3969515

[8] Mignone JL , Roig‐Lopez JL , Fedtsova N , et al. Neural potential of a stem cell population in the hair follicle. Cell Cycle. 2007;6(17):2161‐2170. 17873521 10.4161/cc.6.17.4593 PMC3789384

[9] Amoh Y , Li L , Katsuoka K , Penman S , Hoffman RM . Multipotent nestin‐positive, keratin‐negative hair‐follicle bulge stem cells can form neurons. Proc Natl Acad Sci U S A. 2005;102(15):5530‐5534. 15802470 10.1073/pnas.0501263102 PMC556262

[10] Yamane M , Takaoka N , Obara K , et al. Hair‐follicle‐associated pluripotent (HAP) stem cells can extensively differentiate to tyrosine‐hydroxylase‐expressing dopamine‐secreting neurons. Cell. 2021;10(4):864. 10.3390/cells10040864 PMC8069047 33920157

[11] Gho CG , Schomann T , de Groot SC , et al. Isolation, expansion and neural differentiation of stem cells from human plucked hair: a further step towards autologous nerve recovery. Cytotechnology. 2016;68(5):1849‐1858. 26702932 10.1007/s10616-015-9938-x PMC5023559

[12] Sakaue M , Sieber‐Blum M . Human epidermal neural crest stem cells as a source of Schwann cells. Development. 2015;142(18):3188‐3197. 26251357 10.1242/dev.123034 PMC4582175

[13] Pournajaf S , Valian N , Shalmani LM , Khodabakhsh P , Jorjani M , Dargahi L . Fingolimod increases oligodendrocytes markers expression in epidermal neural crest stem cells. Eur J Pharmacol. 2020;885:173502. 32860811 10.1016/j.ejphar.2020.173502

[14] Pandamooz S , Salehi MS , Zibaii MI , Ahmadiani A , Nabiuni M , Dargahi L . Epidermal neural crest stem cell‐derived glia enhance neurotrophic elements in an ex vivo model of spinal cord injury. J Cell Biochem. 2018;119(4):3486‐3496. 29143997 10.1002/jcb.26520

[15] Pandamooz S , Salehi MS , Safari A , et al. Enhancing the expression of neurotrophic factors in epidermal neural crest stem cells by valproic acid: a potential candidate for combinatorial treatment. Neurosci Lett. 2019;704:8‐14. 30904572 10.1016/j.neulet.2019.03.033

[16] Hu YF , Gourab K , Wells C , Clewes O , Schmit BD , Sieber‐Blum M . Epidermal neural crest stem cell (EPI‐NCSC)—mediated recovery of sensory function in a mouse model of spinal cord injury. Stem Cell Rev Rep. 2010;6(2):186‐198. 20414748 10.1007/s12015-010-9152-3 PMC2887506

[17] Shalmani LM , Valian N , Pournajaf S , Abbaszadeh F , Dargahi L , Jorjani M . Combination therapy with astaxanthin and epidermal neural crest stem cells improves motor impairments and activates mitochondrial biogenesis in a rat model of spinal cord injury. Mitochondrion. 2020;52:125‐134. 32151747 10.1016/j.mito.2020.03.002

[18] Obara K , Shirai K , Hamada Y , et al. Chronic spinal cord injury functionally repaired by direct implantation of encapsulated hair‐follicle‐associated pluripotent (HAP) stem cells in a mouse model: potential for clinical regenerative medicine. Plos One. 2022;17(1):e0262755. 35085322 10.1371/journal.pone.0262755 PMC8794105

[19] Amoh Y , Li L , Campillo R , et al. Implanted hair follicle stem cells form Schwann cells that support repair of severed peripheral nerves. Proc Natl Acad Sci U S A. 2005;102(49):17734‐17738. 16314569 10.1073/pnas.0508440102 PMC1308908

[20] Lin H , Liu F , Zhang C , et al. Pluripotent hair follicle neural crest stem‐cell‐derived neurons and schwann cells functionally repair sciatic nerves in rats. Mol Neurobiol. 2009;40(3):216‐223. 19728182 10.1007/s12035-009-8082-z

[21] Zhang L , Li B , Liu B , Dong Z . Co‐transplantation of epidermal neural crest stem cells and olfactory ensheathing cells repairs sciatic nerve defects in rats. Front Cell Neurosci. 2019;13:253. 31244611 10.3389/fncel.2019.00253 PMC6582070

[22] Salehi MS , Pandamooz S , Safari A , et al. Epidermal neural crest stem cell transplantation as a promising therapeutic strategy for ischemic stroke. CNS Neurosci Ther. 2020;26(7):670‐681. 32281225 10.1111/cns.13370 PMC7298983

[23] Mousavi SM , Akbarpour B , Karimi‐Haghighi S , et al. Therapeutic potential of hair follicle‐derived stem cell intranasal transplantation in a rat model of ischemic stroke. BMC Neurosci. 2022;23(1):1‐14. 35879657 10.1186/s12868-022-00732-w PMC9316709

[24] Moeinabadi‐Bidgoli K , Babajani A , Yazdanpanah G , et al. Translational insights into stem cell preconditioning: from molecular mechanisms to preclinical applications. Biomed Pharmacother. 2021;142:112026. 34411911 10.1016/j.biopha.2021.112026

[25] Liu S , Zhou J , Zhang X , et al. Strategies to optimize adult stem cell therapy for tissue regeneration. Int J Mol Sci. 2016;17(6):982. 27338364 10.3390/ijms17060982 PMC4926512

[26] Zhao L , Hu C , Han F , Cai F , Wang J , Chen J . Preconditioning is an effective strategy for improving the efficiency of mesenchymal stem cells in kidney transplantation. Stem Cell Res Ther. 2020;11:1‐11. 32448356 10.1186/s13287-020-01721-8 PMC7245776

[27] Zhang S , Lachance BB , Moiz B , Jia X . Optimizing stem cell therapy after ischemic brain injury. J Stroke. 2020;22(3):286‐305. 33053945 10.5853/jos.2019.03048 PMC7568970

[28] Zhu S‐z , Szeto V , Bao M‐h , Sun H‐s , Feng Z‐p . Pharmacological approaches promoting stem cell‐based therapy following ischemic stroke insults. Acta Pharmacol Sin. 2018;39(5):695‐712. 29671416 10.1038/aps.2018.23 PMC5943911

[29] Shafiq M , Jung Y , Kim SH . Insight on stem cell preconditioning and instructive biomaterials to enhance cell adhesion, retention, and engraftment for tissue repair. Biomaterials. 2016;90:85‐115. 27016619 10.1016/j.biomaterials.2016.03.020

[30] Lee B‐C , Kang K‐S . Functional enhancement strategies for immunomodulation of mesenchymal stem cells and their therapeutic application. Stem Cell Res Ther. 2020;11(1):1‐10. 32928306 10.1186/s13287-020-01920-3 PMC7491075

[31] Salehi MS , Safari A , Pandamooz S , et al. The beneficial potential of genetically modified stem cells in the treatment of stroke: a review. Stem Cell Rev Rep. 2022;18(2):412‐440. 34033001 10.1007/s12015-021-10175-1 PMC8144279

[32] de Cássia NN , Mizukami A , Caliári‐Oliveira C , et al. Priming approaches to improve the efficacy of mesenchymal stromal cell‐based therapies. Stem Cell Res Ther. 2019;10(1):1‐21. 31046833 10.1186/s13287-019-1224-y PMC6498654

[33] Pandamooz S , Jafari A , Salehi MS , et al. Substrate stiffness affects the morphology and gene expression of epidermal neural crest stem cells in a short term culture. Biotechnol Bioeng. 2020;117(2):305‐317. 31654402 10.1002/bit.27208

[34] Hu YF , Zhang ZJ , Sieber‐Blum M . An epidermal neural crest stem cell (EPI‐NCSC) molecular signature. Stem Cells. 2006;24(12):2692‐2702. 16931771 10.1634/stemcells.2006-0233

[35] Pajtler K , Bohrer A , Maurer J , et al. Production of chick embryo extract for the cultivation of murine neural crest stem cells. J Vis Exp. 2010;45:2380. 10.3791/2380 PMC3159602 21178955

[36] Sieber‐Blum M , Grim M . The adult hair follicle: cradle for pluripotent neural crest stem cells. Birth Defects Res C Embryo Today. 2004;72(2):162‐172. 15269890 10.1002/bdrc.20008

[37] Pandamooz S , Naji M , Alinezhad F , Zarghami A , Pourghasem M . The influence of cerebrospinal fluid on epidermal neural crest stem cells may pave the path for cell‐based therapy. Stem Cell Res Ther. 2013;4(4):1‐9. 23867009 10.1186/scrt235 PMC3854676

[38] Sieber‐Blum M , Grim M , Hu Y , Szeder V . Pluripotent neural crest stem cells in the adult hair follicle. Dev Dyn. 2004;231(2):258‐269. 15366003 10.1002/dvdy.20129

[39] Salehi MS , Neumann ID , Jurek B , Pandamooz S . Co‐stimulation of oxytocin and arginine‐vasopressin receptors affect hypothalamic neurospheroid size. Int J Mol Sci. 2021;22(16):8464. 34445168 10.3390/ijms22168464 PMC8395152

[40] Fischer R , Kessler BM . Gel‐aided sample preparation (GASP)—a simplified method for gel‐assisted proteomic sample generation from protein extracts and intact cells. Proteomics. 2015;15(7):1224‐1229. 25515006 10.1002/pmic.201400436 PMC4409837

[41] Ma J , Guo Y , Hu J , et al. The positive effect of chick embryo and nutrient mixture on bone marrow‐derived mesenchymal stem cells from aging rats. Sci Rep. 2018;8(1):1‐11. 29728592 10.1038/s41598-018-25563-w PMC5935737

[42] Karunarathne WK , Giri L , Kalyanaraman V , Gautam N . Optically triggering spatiotemporally confined GPCR activity in a cell and programming neurite initiation and extension. Proc Natl Acad Sci U S A. 2013;110(17):E1565‐E1574. 23479634 10.1073/pnas.1220697110 PMC3637763

[43] Lestanova Z , Bacova Z , Bakos J . Mechanisms involved in the regulation of neuropeptide‐mediated neurite outgrowth: a minireview. Endocr Regul. 2016;50(2):72‐82. 27560639 10.1515/enr-2016-0011

[44] Jana M , Pahan K . Astrocytes, oligodendrocytes and schwann cells. Neuroimmune Pharmacology. Springer; 2017:117‐140.

[45] Jessen KR , Mirsky R , Lloyd AC . Schwann cells: development and role in nerve repair. Cold Spring Harb Perspect Biol. 2015;7(7):a020487. 25957303 10.1101/cshperspect.a020487 PMC4484967

[46] Britsch S , Goerich DE , Riethmacher D , et al. The transcription factor Sox10 is a key regulator of peripheral glial development. Genes Dev. 2001;15(1):66‐78. 11156606 10.1101/gad.186601 PMC312607

[47] Yan X , Liu J , Ye Z , et al. CaMKII‐mediated CREB phosphorylation is involved in Ca2+−induced BDNF mRNA transcription and neurite outgrowth promoted by electrical stimulation. PLoS One. 2016;11(9):e0162784. 27611779 10.1371/journal.pone.0162784 PMC5017744

[48] Jeon S‐H , Chae B‐C , Kim H‐A , et al. The PKA/CREB pathway is closely involved in VEGF expression in mouse macrophages. Mol Cells. 2007;23(1):23‐29. 17464208

[49] Ibrahim SAE‐F , Abudu A , Jonhson E , Aftab N , Conrad S , Fluck M . The role of AP‐1 in self‐sufficient proliferation and migration of cancer cells and its potential impact on an autocrine/paracrine loop. Oncotarget. 2018;9(76):34259‐34278. 30344941 10.18632/oncotarget.26047 PMC6188139

[50] Kang Y , Patel NR , Shively C , et al. Dual threshold optimization and network inference reveal convergent evidence from TF binding locations and TF perturbation responses. Genome Res. 2020;30(3):459‐471. 32060051 10.1101/gr.259655.119 PMC7111528

[51] McKelvey KJ , Powell KL , Ashton AW , Morris JM , McCracken SA . Exosomes: mechanisms of uptake. J Circ Biomark. 2015;4:7. 28936243 10.5772/61186 PMC5572985

[52] Ferrer E , Dunmore BJ , Hassan D , et al. A potential role for exosomal translationally controlled tumor protein export in vascular remodeling in pulmonary arterial hypertension. Am J Respir Cell Mol Biol. 2018;59(4):467‐478. 29676587 10.1165/rcmb.2017-0129OC PMC6178156

[53] Pinkaew D , Fujise K . Fortilin: a potential target for the prevention and treatment of human diseases. Adv Clin Chem. 2017;82:265‐300. 28939212 10.1016/bs.acc.2017.06.006 PMC6064975

[54] Bommer U‐A . The translational controlled tumour protein TCTP: biological functions and regulation. Results Probl Cell Differ. 2017;64:69‐126. 29149404 10.1007/978-3-319-67591-6_4

[55] Chen S‐H , Lu C‐H , Tsai M‐J . TCTP is essential for cell proliferation and survival during CNS development. Cell. 2020;9(1):133. 10.3390/cells9010133 PMC7017002 31935927

[56] Roque CG , Holt CE . TCTP in neuronal circuitry assembly. TCTP/tpt1‐Remodeling Signaling from Stem Cell to Disease. Springer; 2017:201‐215. 10.1007/978-3-319-67591-6_10 29149410

[57] Jimenez CR , Stam FJ , Li KW , et al. Proteomics of the injured rat sciatic nerve reveals protein expression dynamics during regeneration. Mol Cell Proteomics. 2005;4(2):120‐132. 15509515 10.1074/mcp.M400076-MCP200

[58] Ren J , Mao X , Chen M , et al. TCTP expression after rat spinal cord injury: implications for astrocyte proliferation and migration. J Mol Neurosci. 2015;57(3):366‐375. 26266488 10.1007/s12031-015-0628-0

[59] Amzallag N , Passer BJ , Allanic D , et al. TSAP6 facilitates the secretion of translationally controlled tumor protein/histamine‐releasing factor via a nonclassical pathway. J Biol Chem. 2004;279(44):46104‐46112. 15319436 10.1074/jbc.M404850200

[60] Fotovati A , Abu‐Ali S , Wang P‐S , et al. YB‐1 bridges neural stem cells and brain tumor–initiating cells via its roles in differentiation and cell growth. Cancer Res. 2011;71(16):5569‐5578. 21730024 10.1158/0008-5472.CAN-10-2805

[61] Frye BC , Halfter S , Djudjaj S , et al. Y‐box protein‐1 is actively secreted through a non‐classical pathway and acts as an extracellular mitogen. EMBO Rep. 2009;10(7):783‐789. 19483673 10.1038/embor.2009.81 PMC2690452

[62] Sundqvist A , Zieba A , Vasilaki E , et al. Specific interactions between Smad proteins and AP‐1 components determine TGFβ‐induced breast cancer cell invasion. Oncogene. 2013;32(31):3606‐3615. 22926518 10.1038/onc.2012.370

[63] Shelke GV , Yin Y , Jang SC , et al. Endosomal signalling via exosome surface TGFβ‐1. J Extracell Vesicles. 2019;8(1):1650458. 31595182 10.1080/20013078.2019.1650458 PMC6764367

[64] Achilleos A , Trainor PA . Neural crest stem cells: discovery, properties and potential for therapy. Cell Res. 2012;22(2):288‐304. 22231630 10.1038/cr.2012.11 PMC3271580

[65] Gericota B , Anderson JS , Mitchell G , et al. Canine epidermal neural crest stem cells: characterization and potential as therapy candidate for a large animal model of spinal cord injury. Stem Cells Transl Med. 2014;3(3):334‐345. 24443004 10.5966/sctm.2013-0129 PMC3952930

[66] McMahill BG , Spriet M , Sisó S , et al. Feasibility study of canine epidermal neural crest stem cell transplantation in the spinal cords of dogs. Stem Cells Transl Med. 2015;4(10):1173‐1186. 26273065 10.5966/sctm.2015-0018 PMC4572898

[67] Amoh Y , Li L , Katsuoka K , Hoffman RM . Multipotent hair follicle stem cells promote repair of spinal cord injury and recovery of walking function. Cell Cycle. 2008;7(12):1865‐1869. 18583926 10.4161/cc.7.12.6056

[68] Li Y , Yao D , Zhang J , et al. The effects of epidermal neural crest stem cells on local inflammation microenvironment in the defected sciatic nerve of rats. Front Mol Neurosci. 2017;10:133. 28588447 10.3389/fnmol.2017.00133 PMC5438963

[69] Amoh Y , Hamada Y , Aki R , Kawahara K , Hoffman RM , Katsuoka K . Direct transplantation of uncultured hair‐follicle pluripotent stem (hfPS) cells promotes the recovery of peripheral nerve injury. J Cell Biochem. 2010;110(1):272‐277. 20411592 10.1002/jcb.22534

[70] Pringle NP , Yu W‐P , Guthrie S , et al. Determination of neuroepithelial cell fate: induction of the oligodendrocyte lineage by ventral midline cells and sonic hedgehog. Dev Biol. 1996;177(1):30‐42. 8660874 10.1006/dbio.1996.0142

[71] Kalyani A , Hobson K , Rao MS . Neuroepithelial stem cells from the embryonic spinal cord: isolation, characterization, and clonal analysis. Dev Biol. 1997;186(2):202‐223. 9205140 10.1006/dbio.1997.8592

[72] Gharaibeh B , Lu A , Tebbets J , et al. Isolation of a slowly adhering cell fraction containing stem cells from murine skeletal muscle by the preplate technique. Nat Protoc. 2008;3(9):1501‐1509. 18772878 10.1038/nprot.2008.142

[73] Oberley TD , Muth JV , Murphy‐Ullrich J . Growth and maintenance of glomerular cells under defined conditions. Am J Pathol. 1980;101(1):195‐204. 7446700 PMC1903587

[74] Maxwell GD , Reid K , Elefanty A , Bartlett PF , Murphy M . Glial cell line‐derived neurotrophic factor promotes the development of adrenergic neurons in mouse neural crest cultures. Proc Natl Acad Sci U S A. 1996;93(23):13274‐13279. 8917581 10.1073/pnas.93.23.13274 PMC24083

[75] Mohlin S , Kerosuo L . In vitro maintenance of multipotent neural crest stem cells as crestospheres. Stem Cell Niche. Springer; 2018:1‐11. 10.1007/7651_2018_180 PMC8014252 30159826

[76] Kerosuo L , Nie S , Bajpai R , Bronner ME . Crestospheres: long‐term maintenance of multipotent, premigratory neural crest stem cells. Stem Cell Rep. 2015;5(4):499‐507. 10.1016/j.stemcr.2015.08.017 PMC4625028 26441305

[77] Mohlin S , Kunttas E , Persson CU , et al. Maintaining multipotent trunk neural crest stem cells as self‐renewing crestospheres. Dev Biol. 2019;447(2):137‐146. 30664880 10.1016/j.ydbio.2019.01.010 PMC6497816

[78] Christman S , Kong B , Landry M , Foster D . Chicken embryo extract mitigates growth and morphological changes in a spontaneously immortalized chicken embryo fibroblast cell line. Poult Sci. 2005;84(9):1423‐1431. 16206564 10.1093/ps/84.9.1423

[79] Bałaban J , Wierzbicki M , Zielińska M , et al. Effects of graphene oxide nanofilm and chicken embryo muscle extract on muscle progenitor cell differentiation and contraction. Molecules. 2020;25(8):1991. 32340398 10.3390/molecules25081991 PMC7221809

[80] Sieber‐Blum M . Human epidermal neural crest stem cells as candidates for cell‐based therapies, disease modeling, and drug discovery. Birth Defects Res C Embryo Today. 2014;102(3):221‐226. 25228472 10.1002/bdrc.21073

[81] Ohyama M , Terunuma A , Tock CL , et al. Characterization and isolation of stem cell–enriched human hair follicle bulge cells. J Clin Invest. 2006;116(1):249‐260. 16395407 10.1172/JCI26043 PMC1323261

[82] Vasyliev RG , Gubar OS , Gordiienko IM , et al. Comparative analysis of biological properties of large‐scale expanded adult neural crest‐derived stem cells isolated from human hair follicle and skin dermis. Stem Cells Int. 2019;2019:1‐20. 10.1155/2019/9640790 PMC6399535 30915126

[83] Lange C , Storkebaum E , De Almodóvar CR , Dewerchin M , Carmeliet P . Vascular endothelial growth factor: a neurovascular target in neurological diseases. Nat Rev Neurol. 2016;12(8):439‐454. 27364743 10.1038/nrneurol.2016.88

[84] Ma Y , Zechariah A , Qu Y , Hermann DM . Effects of vascular endothelial growth factor in ischemic stroke. J Neurosci Res. 2012;90(10):1873‐1882. 22714747 10.1002/jnr.23088

[85] Autry AE , Monteggia LM . Brain‐derived neurotrophic factor and neuropsychiatric disorders. Pharmacol Rev. 2012;64(2):238‐258. 22407616 10.1124/pr.111.005108 PMC3310485

[86] Giacobbo BL , Doorduin J , Klein HC , Dierckx RA , Bromberg E , de Vries EF . Brain‐derived neurotrophic factor in brain disorders: focus on neuroinflammation. Mol Neurobiol. 2019;56(5):3295‐3312. 30117106 10.1007/s12035-018-1283-6 PMC6476855

[87] Zuccato C , Cattaneo E . Brain‐derived neurotrophic factor in neurodegenerative diseases. Nat Rev Neurol. 2009;5(6):311‐322. 19498435 10.1038/nrneurol.2009.54

[88] Scheper V , Schwieger J , Hamm A , Lenarz T , Hoffmann A . BDNF‐overexpressing human mesenchymal stem cells mediate increased neuronal protection in vitro. J Neurosci Res. 2019;97(11):1414‐1429. 31257632 10.1002/jnr.24488 PMC6772136

[89] Kopra JJ , Panhelainen A , Af Bjerkén S , et al. Dampened amphetamine‐stimulated behavior and altered dopamine transporter function in the absence of brain GDNF. J Neurosci. 2017;37(6):1581‐1590. 28096470 10.1523/JNEUROSCI.1673-16.2016 PMC6705679

[90] Finzsch M , Schreiner S , Kichko T , et al. Sox10 is required for Schwann cell identity and progression beyond the immature Schwann cell stage. J Cell Biol. 2010;189(4):701‐712. 20457761 10.1083/jcb.200912142 PMC2872908

[91] Khodabakhsh P , Pournajaf S , Mohaghegh Shalmani L , Ahmadiani A , Dargahi L . Insulin promotes Schwann‐like cell differentiation of rat epidermal neural crest stem cells. Mol Neurobiol. 2021;58(10):5327‐5337. 34297315 10.1007/s12035-021-02423-9

[92] Kubiak JZ , Kloc M . Elusive role of TCTP protein and mRNA in cell cycle and cytoskeleton regulation. In: Telerman A, Amson R, eds. TCTP/tpt1‐Remodeling Signaling from Stem Cell to Disease. Cham: Springer International Publishing; 2017:217‐225. 10.1007/978-3-319-67591-6_11 29149411

[93] Yu H , Kumar SM , Kossenkov AV , Showe L , Xu X . Stem cells with neural crest characteristics derived from the bulge region of cultured human hair follicles. J Invest Dermatol. 2010;130(5):1227‐1236. 19829300 10.1038/jid.2009.322 PMC3050599

[94] Lei M , Schumacher LJ , Lai Y‐C , et al. Self‐organization process in newborn skin organoid formation inspires strategy to restore hair regeneration of adult cells. Proc Natl Acad Sci U S A. 2017;114(34):E7101‐E7110. 28798065 10.1073/pnas.1700475114 PMC5576784

[95] Lee J , Rabbani CC , Gao H , et al. Hair‐bearing human skin generated entirely from pluripotent stem cells. Nature. 2020;582(7812):399‐404. 32494013 10.1038/s41586-020-2352-3 PMC7593871

[96] Quadrato G , Brown J , Arlotta P . The promises and challenges of human brain organoids as models of neuropsychiatric disease. Nat Med. 2016;22(11):1220‐1228. 27783065 10.1038/nm.4214

[97] Balakrishnan A , Belfiore L , Chu T‐H , et al. Insights into the role and potential of schwann cells for peripheral nerve repair from studies of development and injury. Front Mol Neurosci. 2021;13:608442. 33568974 10.3389/fnmol.2020.608442 PMC7868393

[98] Chen G , Luo X , Wang W , Wang Y , Zhu F , Wang W . Interleukin‐1β promotes Schwann cells de‐differentiation in Wallerian degeneration via the c‐JUN/AP‐1 pathway. Front Cell Neurosci. 2019;13:304. 31338026 10.3389/fncel.2019.00304 PMC6629865

[99] Ramesh R , Manurung Y , Ma KH , et al. JUN regulation of injury‐induced enhancers in Schwann cells. J Neurosci. 2022;42(34):6506‐6517. 35906072 10.1523/JNEUROSCI.2533-21.2022 PMC9410756

